# Single shot multispectral multidimensional imaging using chaotic waves

**DOI:** 10.1038/s41598-020-70849-7

**Published:** 2020-08-17

**Authors:** Vijayakumar Anand, Soon Hock Ng, Jovan Maksimovic, Denver Linklater, Tomas Katkus, Elena P. Ivanova, Saulius Juodkazis

**Affiliations:** 1 Center for Micro-Photonics, Faculty of Science, Engineering and Technology, Swinburne University of Technology, Hawthorn, VIC 3122 Australia; 2 Department of Physics, RMIT, GPO Box 2476, Melbourne, VIC 3001 Australia; 3 Melbourne Centre for Nanofabrication, ANFF, 151 Wellington Road, Clayton, VIC 3168 Australia; 4 Tokyo Tech World Research Hub Initiative (WRHI), School of Materials and Chemical Technology, Tokyo Institute of Technology, 2-12-1, Ookayama, Meguro-ku, Tokyo 152-8550 Japan

**Keywords:** Applied optics, Optical techniques

## Abstract

Multispectral imaging technology is a valuable scientific tool for various applications in astronomy, remote sensing, molecular fingerprinting, and fluorescence imaging. In this study, we demonstrate a single camera shot, lensless, interferenceless, motionless, non-scanning, space, spectrum, and time resolved five-dimensional incoherent imaging technique using tailored chaotic waves with quasi-random intensity and phase distributions. Chaotic waves can distinctly encode spatial and spectral information of an object in single self-interference intensity distribution. In this study, a tailored chaotic wave with a nearly pure phase function and lowest correlation noise is generated using a quasi-random array of pinholes. A unique sequence of signal processing techniques is applied to extract all possible spatial and spectral channels with the least entropy. The depth-wavelength reciprocity is exploited to see colour from depth and depth from colour and the physics of beam propagation is exploited to see at one depth by calibrating at another.

## Introduction

Hyperspectral and multispectral imaging technologies offer the capability to extract spectral information for every pixel of an image and are useful for a wide range of applications ranging from astronomy^1^, biology^2^, fluorescence imaging^3^, infrared imaging^4^, and industrial applications^5,6^. The experimental and computational footprint of the above hyperspectral imagers are enormous owing to the principle of direct imaging. Pinhole imaging is one of the oldest direct imaging technologies developed several centuries ago with the first published report in 1545 showing a drawing in Gemma Frisius’ *De Radio Astronomica et Geometrica*^7^. Pinhole imaging continues to be one of the attractive areas of research as the technique is easier to implement in almost any band of the electromagnetic spectrum^8,9^ and exhibits distortion, astigmatism free and infinite depth of field imaging capabilities^10^. Pinhole imaging systems have been proven useful in astronomical^11^, biomedical applications^12^, and holography^13^. One of the main drawbacks of pinhole imaging is that most of the incoming light is blocked resulting in a low intense image with a poor SNR. Substitutions to pinhole such Fresnel zone plate^14^, photon sieves^15^, random array of pinholes (RAP)^16^, coded apertures^17^, etc., have proven to overcome the above problem.

Indirect imaging techniques such as imaging with RAP and coded apertures were born on occasions when the capabilities of direct imagers were not sufficient to achieve a particular task. For instance, when the pinhole was replaced by a RAP, direct imaging became indirect as what was recorded or observed is not the image of a scene but a random like intensity distribution. Computational processes were often needed to reconstruct the image from the random like intensity distribution. A direct imager has a direct object-image relationship, whereas, indirect imagers have an object-intermediate complex amplitude–image relationship. The existing intermediate step is what makes indirect imagers possess capabilities that are not present in direct imagers. Indirect imaging techniques such as holography in which instead of the image of an object, a hologram of a scene is recorded, offers ample opportunities to extract additional information of a scene than direct imaging^18,19^. However, holography techniques often suffer from penalties paid in the form of many number of camera recordings, and the need for infrastructure for two beam interference such as vibration isolation, coherent light source, many optical elements, etc. which precludes its’ application in industry and extreme environments. The latest advancements in holography technology assisted by computational optical methods^20^ and deep learning modules have enriched the area of research and simplified the imaging processes^21^ by transferring the experimental requirement load partly to signal processing, computational and machine learning domains. This resulted in compact, lensless, multidimensional imaging tools with advanced imaging capabilities on par with or better than expensive direct imaging counterparts.

The complex amplitude generated by a RAP has a random intensity and phase distribution resulting in a chaotic wave. Chaotic waves have been shown recently to possess the capability to encode three-dimensional location information of an object into a random intensity pattern^22^ without the need for two beam interference and spatial scanning like holography and direct imaging respectively. With an appropriate signal processing tool, it is possible to decode depth specific information from one or few intensity patterns. In this study, we synthesize tailored incoherent chaotic waves using a quasi-random array of pinholes (QRAP) to encode spatial as well as spectral information of an object into a single monochrome intensity pattern and decode that intensity pattern into multiple spatial and spectral image channels using unique spatio-spectral library functions. The above process increases the information bandwidth of imaging and videography incredibly. This is in contrast to direct imaging modules, where every spatio-spectral channel is recorded by a scanning procedure. There are certain phase retrieval techniques developed which do not require the spatio-spectral library to extract information from a chaotic wave, but the main problem was that all the spatial and spectral channels were superposed during image reconstruction into a single image^23^.

Unlike direct imaging methods, where any object under observation is spatially and spectrally scanned, the proposed method requires only a one time manual-cum-computer training procedure during which all possible spatio-spectral signatures are synthesized and stored. When a multispectral, thick object is observed using the QRAP, only a single monochrome intensity pattern is needed to decode all the spatio-spectral channels. In the previous studies on diffuser based spatial and spectral cameras, it is necessary to scan over all the spectrum and space^24,25^. Besides, the chaotic waves could not be engineered resulting in a lower field of view of imaging and higher reconstruction noise demanding complicated computational reconstruction mechanisms. Multispectral imaging techniques such as coded aperture snap shot spectral imaging (CASSI) and compressive spectral imaging techniques have been useful for reconstructing 3D information of a scene in 2D space and spectrum but requires multiple optical components and could not be used to simultaneously extract depth as well as spectral information^26–29^. Four dimensional imaging in 3D space and spectrum with coded aperture correlation holography had been demonstrated earlier but requires at least 40 camera shots per image^30^. Another incoherent holography technique called Fresnel incoherent correlation holography^3,31–33^ has been extended for 4D imaging using multiplexing method but the method requires 2 N + 1 camera shots, where N is the number of wavelength channels. Besides, in^31–33^, a holographic optical set up is required with polarization multiplexing and phase shifting. Recently, a modified Fresnel incoherent correlation holography has been demonstrated for 4D imaging with a single camera shot^34^ but requires extensive training in the beginning.

In this study, we propose a unique combination of optical and computational techniques to employ only a single element: an amplitude mask containing a QRAP between the object and the sensor for multispectral, multidimensional imaging with a wide field of view and high information bandwidth with a single camera shot. The method can utilize depth-wavelength reciprocity to employ only a single wavelength to synthesize the entire spectral signatures or a single depth and multiple wavelengths to synthesize the entire spatial signatures or synthesize the entire signatures from a couple of signatures recorded at the axial and spectral limits. The proposed method can perform 5D imaging in space, spectrum and time from a single camera shot after pre-training with only a total of four camera shots recorded at the axial and spectral boundaries of the system.

## Results

### Two-dimensional spatial imaging with chaotic waves

The spatial lateral resolution limit of imaging with chaotic waves is like those of direct imaging with 1.22*λu/D* constrained further by the autocorrelation function, where *D* is the diameter of the RAP mask^35^. A detailed study of the theory and the variation of autocorrelation function with the diameter of the pinholes of the RAP is given in the supplementary materials (Sections S1 and S2). The field of view of imaging using a RAP mask can be twice as much as a lens due to the indirect mode of imaging as even a partial random intensity distribution can reconstruct the complete information of the object. More details on the intrinsic enhanced wide field of view with RAP is given in the supplementary materials (Section S3). The light efficiency has been calculated for the RAP mask and compared with a direct imaging system under identical imaging conditions and the former being an indirect imaging method has a higher photon budget requirement as shown in supplementary materials (Section S4). Consequently, the proposed method is susceptible to noise and lower temporal resolution owing to the need for a higher exposure time. While the principles described above apply to any chaotic wave and it should be possible to image an object with any chaotic wave, the imaging characteristics and performances are dependent upon the nature of chaotic waves. A two layer optimization procedure has been developed to find the optimal locations of the pinholes that will yield a higher signal to noise ratio (SNR). In the first layer, a two-dimensional random variable is iterated several times and the random locations of the pinholes that yielded the maximum SNR is given as input to the second layer. In the second layer, the location of every pinhole was shifted along the *x* and *y* directions by a certain number of pixels and the SNR is calculated for every iteration. The process is repeated for every pinhole and the pinhole locations for the maximum SNR is determined. A detailed optimization procedure to tailoring a chaotic wave by modifying a RAP to a QRAP is given in the supplementary materials (Section S5). In addition to the optimization of the location of pinholes, an adaptive computer processing technique called as non-linear correlation was implemented instead of the conventional correlation methods such as a matched filter or a phase-only filter^36^ to improve the signal to noise ratio along with a median filter and a correlation filter in the spectrum domain. Additional information about the non-linear correlation and adaptability evaluations are given in the supplementary materials (Section S6).

**Figure 1:**
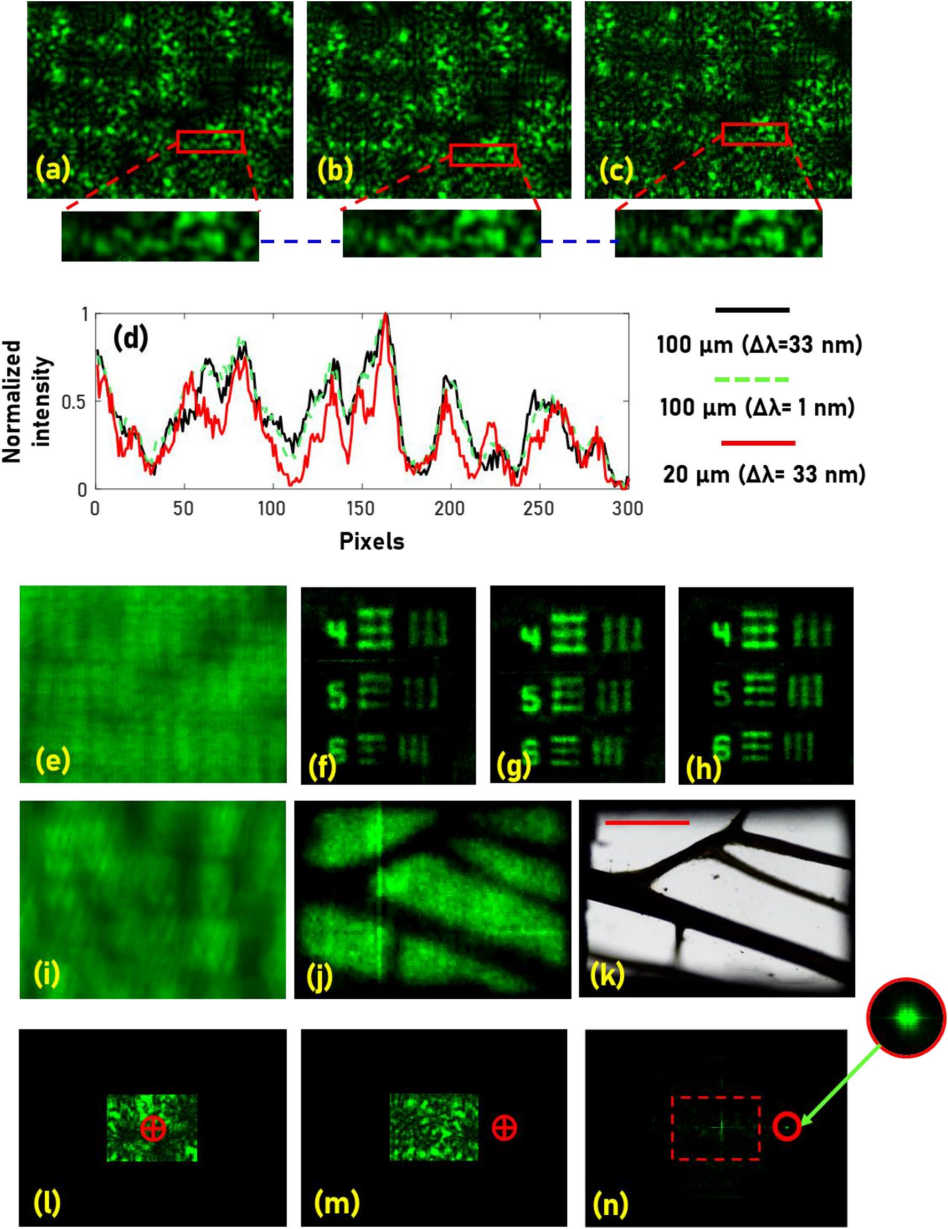
Two-dimensional imaging results. *I*_*PSF*_s for pinholes and spectrum (**a**) 100 μm (Δ*λ* = 33 nm), (**b**) 100 μm (Δ*λ* = 1 nm), (**c**) 20 μm (Δ*λ* = 33 nm). (**d**) Identical signatures in (**a**–**c**) in a line is plotted in black, dotted green and red lines respectively. (**e**) *I*_*O*_ recorded for the USAF object with spectral filter. The results of non-linear correlation (*α* = 0, *β* = 0.6) (**f**) between (**e**) and (**a**), (**g**) between (**e**) and (**b**) and, (**h**) between (**e**) and (**c**). (**i**) *I*_*O*_ recorded for an area of insect wing and (**j**) reconstruction result. (**k**) The image of a wing from a regular microscope with ×5 magnification. (**l**) *I*_*PSF*_ recorded only by the central area (1/3rd) of the image sensor, (**m**) random intensity distribution recorded after the pinhole is shifted in the object plane to move the direct image beyond the field of view limit of direct imaging. (**n**) Reconstruction result of the non-linear correlation between (**i**) and (**j**). The red dotted lines in (**n**) indicates the boundary of the field of view. The red marker in (**l**) and (**m**) indicates the location of the direct image. The blue dotted line indicates the line data region used for comparison in (**d**).

The experiment was carried out using a green LED (*λ*_*c*_ = 530 nm, FWHM = 33 nm), pinholes with diameters 100 μm and 20 μm, USAF object (Group 2, Elements 4, 5 and 6), a 1 nm bandpass filter at 532 nm and a monochrome image sensor. The random intensity distributions for a pinhole with a diameter of 100 μm with a broadband illumination (33 nm), narrow illumination (1 nm) and a pinhole with a diameter of 20 μm without a chromatic filter, for magnification of 0.5 are shown in Fig. 1a–c, respectively. The plots of line data extracted from the random intensity distributions for the above three cases are shown in Fig. 1d. From the plot, it is observed that additional features were visible with 20 μm with higher visibility in comparison to 100 μm pinhole with and without a chromatic filter. The intensity distribution of the USAF object with a chromatic filter is shown in Fig. 1e. The reconstruction results of USAF object for pinholes with 100 μm without and with filter and 20 μm without a filter are shown in Fig. 1f–h respectively. An area of a wing of an insect was imaged using the proposed method. The intensity distribution recorded by the camera, reconstructed image and the same area recorded by a microscope (5×) is shown in Fig. 1i–k, respectively. The wide field of view capability is demonstrated by the following experiment. The image sensor’s central area (~ 1/3rd) was only used for imaging while the rest of the sensor plane was blocked and the pinhole in the object plane was shifted laterally until the direct image is formed beyond the sensor area. The random intensity distributions at the initial and final position of the pinhole are shown in Fig. 1l,m. The reconstruction result beyond the field of view limit of direct imaging is shown in Fig. 1n.

### Three-dimensional spatial imaging with chaotic waves

**Figure 2:**
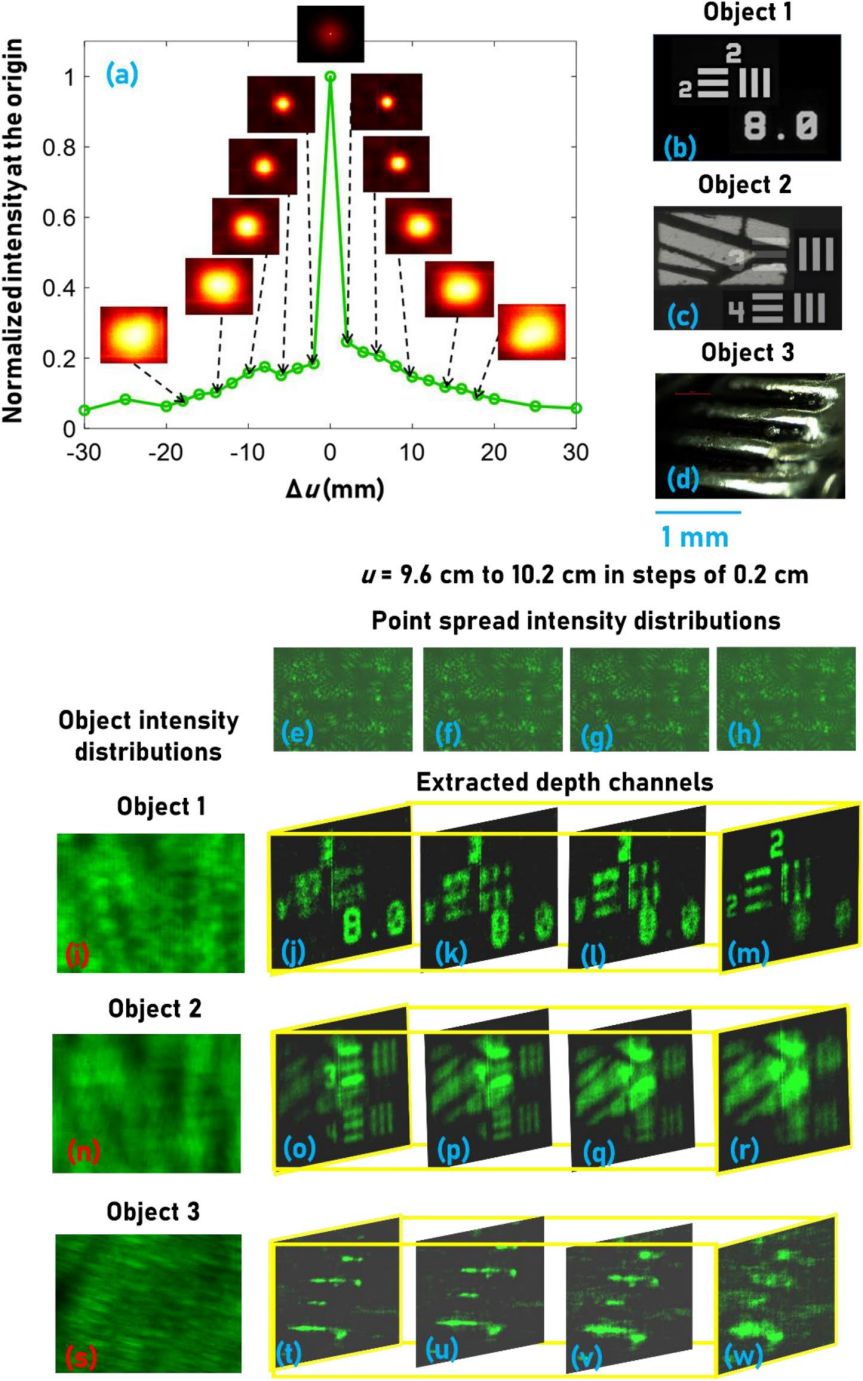
Three-dimensional spatial imaging results. (**a**) Plot of cross-correlation intensity values at the origin with the variation in the axial location of the pinhole. Thick objects composed of (**b**) NBS (8 lp/mm) and USAF (Group 2, Element 2) resolution targets, (**c**) USAF resolution target (Group 2, Elements 3 and 4) and wing of an insect, (**d**) reflective metal strip. Images of the *I*_*PSF*_s recorded for (**e**) *u* = 9.6 cm, (**f**) *u* = 9.8 cm, (**g**) *u* = 10 cm, and (**h**) *u* = 10.2 cm. (**i**) *I*_*O*_ for the thick object (**b**) with a thickness of 6 mm. (**j**–**m**) Depth channels extracted from (**i**) using the *I*_*PSF*_ library (**e**–**h**) respectively. (**n**) *I*_*O*_ recorded for the thick object (**c**) with a thickness of 4 mm. (**o**–**r**) Depth channels extracted from (n) using the *I*_*PSF*_ library (**e**–**h**) respectively. (**s**) *I*_*O*_ recorded for the thick object (**d**) with a thickness of 1 mm. (**t**–**w**) Depth channels extracted from (**s**) using the *I*_*PSF*_ library (**e**–**h**), respectively.

Chaotic waves have axial resolution limit like that of direct imaging given by 8λ(*u*/*D*)^2^. A detailed analysis of the variation of axial resolving power with the diameter of the pinholes is given in the supplementary materials (Section S7). The axial resolution of the system was studied by cross-correlating the *I*_*PSF*_(*u* = 10 cm, *λ*_1_ = 530 nm) with *I*_*PSF*_(Δ*u* = − 3.5 cm to + 3.5 cm, λ_1_ = 530 nm) and the plot of the normalized *I*_*R*_(*x* = 0, *y* = 0) for different values of Δ*u* is shown in Fig. 2a. Different thick objects were constructed using different thin objects: Number ‘8’ of element (8 lp/mm) of NBS target and USAF object (Group 2, Element 2) (Fig. 2b), wings of an insect and USAF object (Group 2, Element 3 and 4) (Fig. 2c) with a spacing of 6 mm and 4 mm respectively. A metallic strip shown in Fig. 2d is the third thick object with a thickness of 2 mm. The *I*_*PSF*_s for *u* = 9.6–10.2 cm in steps of 0.2 cm are shown in Fig. 2e–h, respectively. The *I*_*O*_s of the thick objects Fig. 2b–d, were recorded as shown in Fig. 2i,n,s, respectively. The images of the depth channels extracted from the intensity distributions of the above thick objects using the *I*_*PSF*_s are shown in Fig. 2j–m,o–r,t–w, respectively. The thick object shown in Fig. 2d was illuminated in reflection mode while the other objects were illuminated in transmission mode.

### Three-dimensional spatial and spectral imaging with chaotic waves

**Figure 3:**
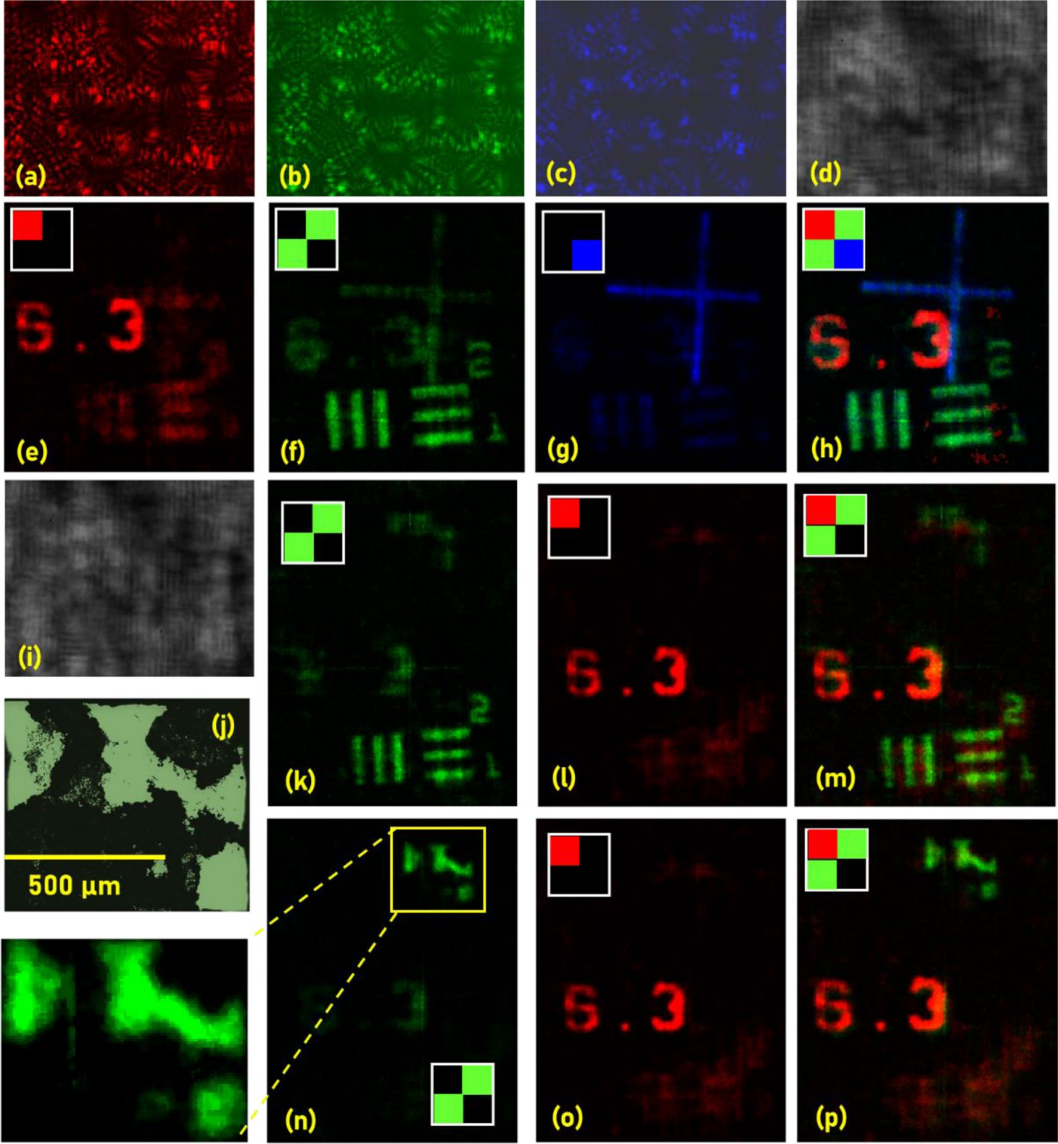
Two-dimensional spatial and spectral imaging results. *I*_*PSF*_s recorded for (**a**) λ = 617 nm, (**b**) 530 nm and (**c**) 488 nm. (**d**) *I*_*O*_ recorded when three thin objects namely NBS (6.3 lp/mm), USAF (Group 2, Element 1) and a negative cross hair located at the same axial distance from the QRAP mask were simultaneously illuminated by red (617 nm), green (530 nm) and blue (488 nm) incoherent light sources respectively. Images of the extracted (**e**) red channel, (**f**) green channel and (**g**) blue channel with a synthetic Bayer mosaic configuration. (**h**) The synthetic multicolour image of the three objects. (**i**) *I*_*O*_ for USAF object and Fungi sample separated by 10 mm illuminated by λ = 530 nm and NBS object located at 3 mm from the Fungi sample illuminated by λ = 617 nm. (**j**) Optical microscope image of the Fungi sample. (**k**) Green channel extracted at the depth of USAF object. (**l**) Red channel extracted at the depth of NBS object. (**m**) Synthetic multicolour image of NBS and USAF object. (**n**) Green channel extracted at the depth of Fungi sample. (**o**) Red channel extracted at the depth of NBS object. (**p**) Synthetic multicolour of Fungi sample and NBS object.

The *I*_*PSF*_s recorded using a pinhole with a diameter of 20 µm for *λ* = 617 nm, 530 nm, and 488 nm are shown in Fig. 3a–c, respectively. Three thin objects namely NBS (6.3 lp/mm), USAF (Group 2, Element 1) and a negative cross hair located at the same axial distance from the QRAP mask were illuminated by red (617 nm), green (530 nm) and blue (488 nm) incoherent light sources respectively. The recorded monochrome random intensity distribution is shown in Fig. 3d. The three spectral channels were extracted from the monochrome intensity distribution by computer processing of the monochrome intensity pattern by the *I*_*PSF*_s. The spectral resolution analysis is presented in the supplementary materials (Section S8). Three matrices were synthesized with a size four times as that of the reconstructed matrix to allow a synthetic Bayer filter mosaic and the extracted three spectral channels were introduced at the appropriate pixels respectively as shown in Fig. 3e–g. The three spectral channels were combined into a single synthetic multicolour image as shown in Fig. 3h. The experiment is repeated to demonstrate five-dimensional imaging using three objects: Fungi sample, USAF and NBS objects. The Fungi sample and USAF object were illuminated by green light while the NBS object was illuminated using red light. The USAF object and Fungi sample was separated by 10 mm from one another and the Fungi sample is located at a distance of 3 mm from the NBS object. The image of the monochrome intensity distribution recorded for the three objects is shown in Fig. 3i. The optical microscope image of the Fungi sample is shown in Fig. 3j. The green and red channels in the synthetic Bayer mosaic extracted at the depth of the USAF object and NBS object are shown in Fig. 3k,l, respectively. The synthetic multicolour image is shown in Fig. 3m. The green and red channels in the synthetic Bayer mosaic extracted at the depth of the Fungi sample and NBS object are shown in Fig. 3n,o, respectively. The synthetic multicolour image is shown in Fig. 3p.

### Depth-wavelength reciprocity

**Figure 4:**
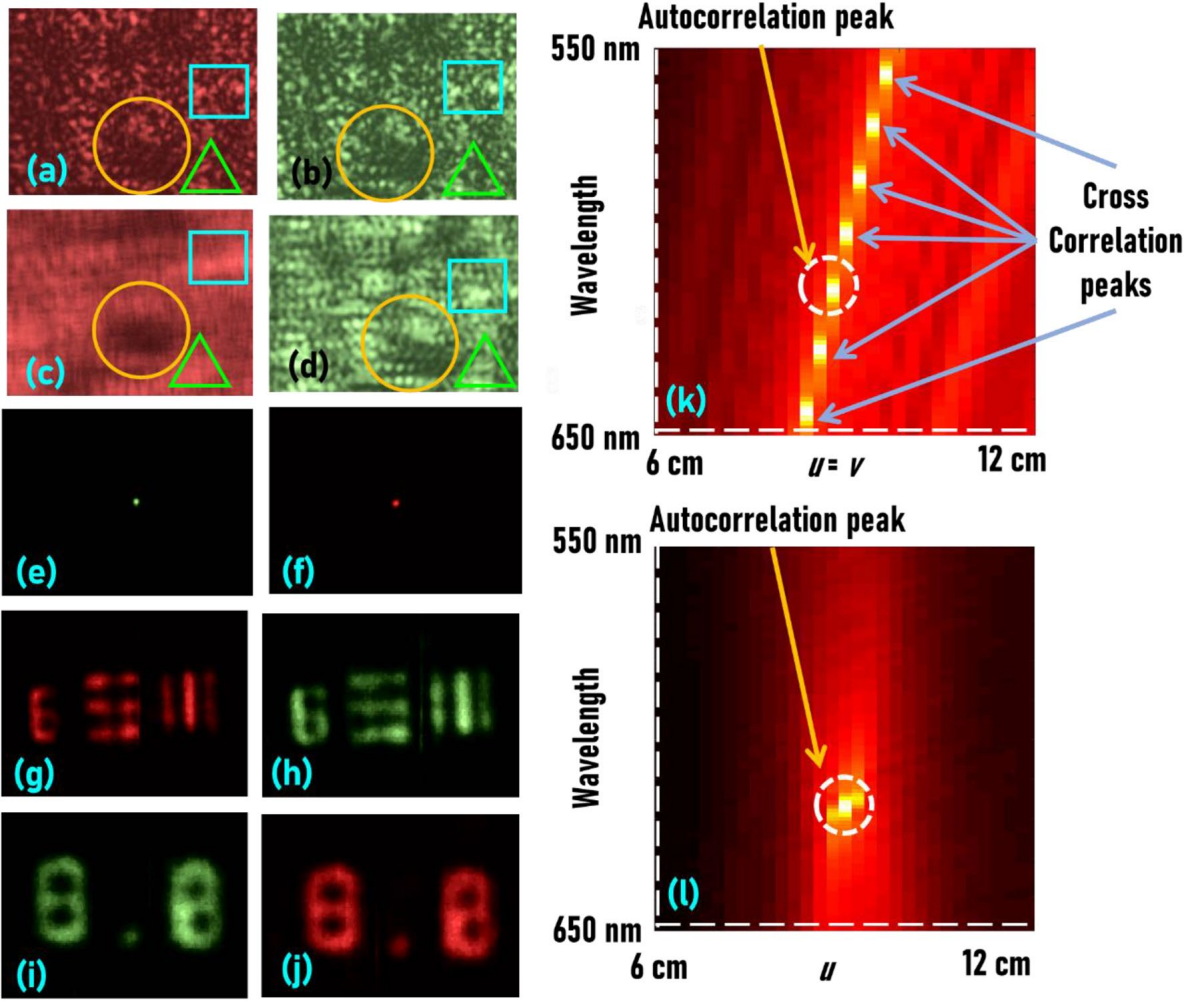
Reconstruction results using depth-wavelength relationship. (**a**) *I*_*PSF*_(*λ* = 617 nm, *u* = *v* = 10 cm), (**b**) *I*_*PSF*_(*λ* = 530 nm, *u* = *v *≈ 12 cm). (**c**) *I*_*O*_(*λ* = 617 nm, *u* = *v* = 10 cm), (**d**) *I*_*O*_(*λ* = 530 nm, *u* = *v *≈ 12 cm). (**e**) Cross-correlation between (**a**) and (**b**). (**f**) Cross-correlation between *I*_*PSF*_(*λ* = 617 nm, *u* = *v *≈ 9 cm) and *I*_*PSF*_(*λ* = 530 nm, *u* = *v* = 10 cm). Reconstruction results by cross-correlating (**g**) (**c**) with (**a**), and (**h**) (**c**) with (**b**), (**i**) (**d**) with (**b**), and (**j**) (**d**) with (**a**). The square, circle and triangle represent similar intensity signatures. (**k**) The object and image distances are varied from 6 to 12 cm and the wavelength was varied from 550 to 650 nm and the corresponding spatio-spectral signatures are cross-correlated with *I*_*PSF*_(*λ* = 617 nm, *u* = *v* = 10 cm). (**l**) The object distance is varied from 6 to 12 cm and the wavelength was varied from 550 to 650 nm and the corresponding spatio-spectral signature is cross-correlated with *I*_*PSF*_(*λ* = 617 nm, *u* = *v* = 10 cm) resulting in a single maxima.

The depth-wavelength relationship is evaluated by the following experiment to see colour from depth and depth from colour (Supplementary section S9). The point spread intensity distribution and object intensity distribution were recorded for the initial values (*u* = *v* = 10 cm) using the red LED (λ = 617 nm) first and the green LED (λ = 530 nm) next. Then in the alternative channels, the *u* and *v* values were adjusted to ≈ (617/530)*u* and (617/530)*v*, and (530/617)*u* and (530/617)*v* and the point spread intensity distributions were recorded using the green LED (λ = 530 nm) and red LED (λ = 617 nm), respectively. As expected, the point spread intensity distribution of the green LED recovered the object information recorded using the red LED and vice versa. The images of the *I*_*PSF*_s (λ = 617 nm, *u* = *v* = 10 cm) and (λ = 530 nm, *u* = *v *≈ 12 cm) are shown in Fig. 4a,b, respectively. The images of the object intensity distributions (Number 8.0 of the element 8 lp/mm of the NBS object and element 6 of group 2 of USAF object) *I*_O_(λ = 617 nm, *u* = *v* = 10 cm) and *I*_*O*_(λ = 530 nm, *u* = *v *≈ 12 cm) are shown in Fig. 4c,d, respectively. The results of cross-correlation between *I*_*PSF*_(λ = 617 nm, *u* = *v* = 10 cm) and *I*_*PSF*_(λ = 530 nm, *u* = *v* ≈ 12 cm) and between *I*_*PSF*_(λ = 617 nm, *u* = *v *≈ 9 cm) and *I*_*PSF*_(λ = 530 nm, *u* = *v* = 10 cm) are shown in Fig. 4e,f, respectively. By comparing Fig. 4a,b, it is possible to even say that both patterns are identical except for a shift. The intensity signatures in different areas denoted by a square, circle and triangle match with one another. Some of the distinct areas are visible in the object intensity pattern as well. The cross-correlation result yields a sharp peak indicating that both Fig. 4a,b are identical. Reconstruction results by cross-correlating Fig. 4c with 4a, and Fig. 4c with 4b, and reconstruction results by cross-correlating Fig. 4d with 4b, and Fig. 4d with 4a are shown in Fig. 4g–j, respectively. The reconstruction results using different wavelength’s point spread functions matches with the reconstruction results with the same wavelength except for some additional background noise and blur. A simulation was carried out with the experimental values of the optical configuration. There are three main parameters namely *u*, *v* and *λ*. The random intensity distribution is calculated for *λ* = 550–650 nm and *u* = *v* = 6–12 cm and cross-correlated for the case with *λ* = 617 nm and *u* = *v* = 10 cm. The plot in Fig. 4k shows the variation of the cross-correlation with respect to change in wavelength and distances. The appearance of multiple peaks with same value indicates that it is possible to generate the same random intensity distribution using different values of *u*, *v* and *λ* due to the depth-wavelength reciprocity given by the equation *λ*_*n*_*u*_*m*_ = *λ*_*n*_*v*_*m*_ = constant. In Fig. 4l, the same simulation has been carried out by keeping *v* as a constant and varying only *u* and *λ*. In this case, there is only one peak which is the autocorrelation peak. This indicates that it is not possible to exploit depth-wavelength reciprocity without changing both *u* and *v* simultaneously. This difference in behaviour reassures that the imaging system is not vulnerable to depth-wavelength ambiguity. The object and image distances and the wavelength are varied and the *I*_*PSF*_ is simulated and cross-correlated with *I*_*PSF*_(*λ* = 617 nm, *u* = *v* = 10 cm) resulting in a generation of a comb of maxima forming at *λ*_*n*_*u*_*m*_ = *λ*_*n*_*v*_*m*_ = constant as shown in Fig. 4k. However, when only the object distance is varied, the comb could not be achieved as shown in Fig. 4l.

### Synthesis of point spread functions

**Figure 5:**
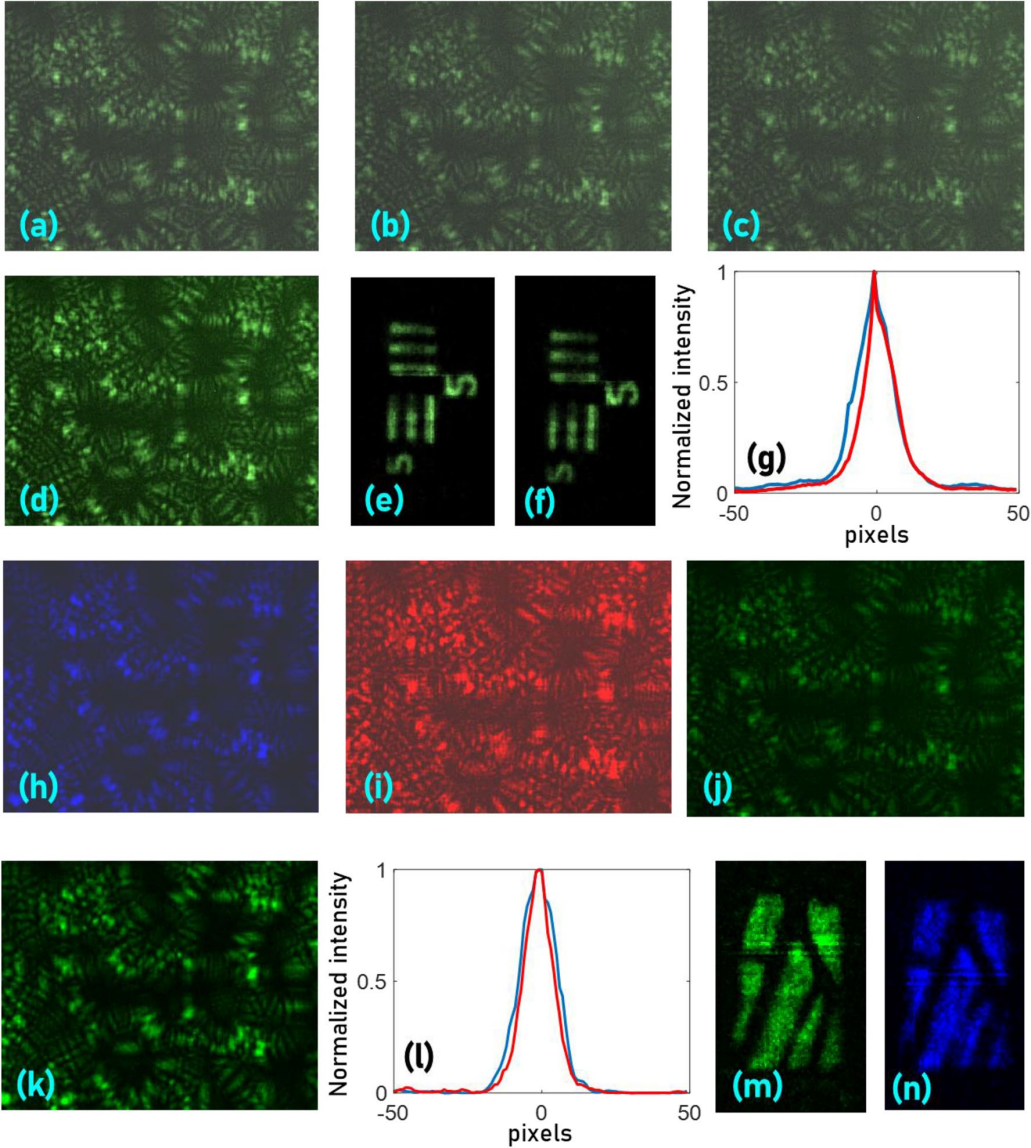
Reconstruction results using synthetic point spread functions. Images of the *I*_*PSF*_s recorded at axial locations (**a**) Δ*u* = − 5 mm, (**b**) Δ*u* = 0 and (**c**) Δ*u* = 5 mm, for λ = 530 nm. (**d**) Image of the *I*_*PSF*_ for Δ*u* = 0, synthesized from Δ*u* = − 5 mm with a magnification factor of 0.9875. Reconstruction results of a USAF object using (**e**) experimentally recorded and (**f**) synthesized point spread function. (**g**) Plots of the cross-correlation functions of the measured *I*_*PSF*_ at Δ*u* = 0 (Red) and the calculated scaled intensity distribution at Δ*u* = -5 mm (Blue) with the measured *I*_*PSF*_ at Δ*u* = 5 mm. Images of the *I*_*PSF*_s recorded at Δ*u* = 0, for (**h**) λ = 488 nm, (**i**) λ = 617 nm and (**j**) λ = 530 nm. (**k**) Image of the *I*_*PSF*_ for λ = 530 nm, synthesized from λ = 488 with a magnification factor of 0.986. (**l**) Plots of the cross-correlation functions of the measured point spread intensity distribution for λ = 530 nm (Red) and the synthesized scaled intensity distribution for λ = 488 nm (Blue) with the measured *I*_*PSF*_ for λ = 617 nm. Reconstruction results of wings of an insect using (**m**) experimentally recorded and (**n**) synthesized *I*_*PSF*_s.

The *I*_*PSF*_s are recorded at axial locations Δ*u* = − 5 mm, 0 and 5 mm, and λ = 530 nm as shown in Fig. 5a–c respectively and the magnification factor γ is measured using the two extreme points as γ = 0.975 and from a linear fit, γ was estimated 0.9875 for the third case (Δ*u* = 0). The image of the synthesized *I*_*PSF*_ for (Δ*u* = 0) is shown in Fig. 5d. The reconstruction results of a USAF object (Group 2, Element 2) using the measured *I*_*PSF*_ for (Δ*u* = 0) and the synthesized *I*_*PSF*_ using the *I*_*PSF*_ recording at Δ*u* = − 5 mm (scaled by 0.9875) are shown in Fig. 5e,f, respectively. The plot of the cross-correlation of the measured *I*_*PSF*_ at Δ*u* = 0 and the calculated scaled intensity distribution at Δ*u* = − 5 mm with the measured *I*_*PSF*_ at Δ*u* = 5 mm are shown in Fig. 5g. The experiment was repeated by recording the *I*_*PSF*_ at Δ*u* = 0 and λ = 488 nm, 530 nm and 617 nm as shown in Fig. 5h–j, respectively. The image of the synthesized *I*_*PSF*_ for the third case λ = 530 nm is shown in Fig. 5k. The magnification factor γ was estimated as 0.96. The plot of the cross-correlation of the measured *I*_*PSF*_ for λ = 530 nm and the synthesized scaled intensity distribution for λ = 488 nm with the measured *I*_*PSF*_ for λ = 617 nm are shown in Fig. 5l. The reconstruction results of the wings of an insect using the measured *I*_*PSF*_ for λ = 530 nm and the synthesized intensity distribution using λ = 488 nm (scaled by 0.9860) are shown in Fig. 5m,n, respectively.

## Discussion

We have proposed and demonstrated a multispectral multidimensional imaging technique using tailored chaotic waves generated by a QRAP. An important property of chaotic waves is the uniformity of the random intensity distribution generated at the sensor plane which in turn controls the background noise during cross-correlation. By tailoring the locations of the pinholes in a mask, it is possible to force a chaotic wave to generate a uniform intensity distribution in the sensor plane. It is well-known that cross-correlation between two positive functions yields higher background noise. Instead of positive functions, if bipolar functions were used, the background noise can be substantially decreased. Intensity patterns recorded by image sensor cannot have negative values and so in practice, two different intensity distributions with cross-correlation value negligible in comparison to autocorrelation were selected and subtracted from one another to generate bipolar intensity patterns. The need for two intensity recordings reduces the temporal resolution of imaging. In this study, a non-linear correlation is applied which tunes the relative magnitudes of the spectrum of the two intensities to produce an effect like that of correlation between two bipolar functions. A sequence of signal processing techniques such as low pass filtering, median filtering, correlation in spectrum domain with optimized values is applied in addition to the non-linear correlation and pinhole array optimization (QRAP) to enhance the signal to noise ratio to the level of direct imaging. The entropy was measured after the above sequence of optical and signal processing techniques to find the recipe for obtaining the highest signal to noise ratio possible.

An important and unidentified property of imaging with a chaotic wave is the possibility of imaging with a wide field of view. While a field of view of about twice as that of a direct imager has been demonstrated in this study (Fig. 1n), in principle, the limit of the field of view can be pushed further by spatial multiplexing schemes^37,38^. In general, the imaging characteristics can be improved by choosing a smaller diameter of pinholes and a narrow spectral width.

The imaging system shown in Fig. 1 can be simulated using mathematical Fresnel propagators which can be decomposed into linear and quadratic phase factors. A closer look into the above forms reveals the intertwined wavelength and depth parameters in the scaling factor *λu* responsible for varying the intensity distributions when the depth or the wavelength is varied. Consequently, the image sensor sees the same change irrespective of whether *λ* is increased or *u* is decreased by the same factor and vice versa. Therefore, to record a random intensity distribution for a different wavelength, it is sufficient to record for a different depth using the same wavelength and vice versa. This property opens numerous possibilities. In other words, by training an imaging system to discriminate information in four dimensions, it can distinctly see information in five dimensions. This is a useful concept as according to the previous studies, to enable spectral sensing in a monochrome camera, a multispectral source is needed, and the recording can be spectrally resolved at those wavelengths only. In the proposed method, the spectrum can be resolved using a monochrome camera and with a single wavelength source for recording the library functions. Alternatively, the advantage can be understood from a different perspective. In the previous studies, to extract information channels from different depths, the library must be recorded at those depths. This is a problem when it comes to imaging through scattering layers. But with the depth-wavelength relationship, a single recording is sufficient at only one plane while the information from other depth channels can be extracted by a mere spectral scanning. We believe that this will be useful to see through scattering layers at different depths. A capability to synthesize the complete library will reduce time and resources during the one-time training procedure. There are some discrepancies in the reconstruction with the synthetic intensity distributions which are partly due to the differences in the spectral widths between the recordings and experimental errors.

There are some challenges associated with the proposed technique. For instance, in a direct imaging system, it is possible to observe an even real-time which is not possible in the proposed technique as only a caustic intensity pattern is observed in this case. A computational assistive module has been provided using open source software (Octave) and operating system (Ubuntu) for real-time observation of events in the supplementary materials (Sections S10 and S11). The lateral, axial and spectral resolutions of indirect imaging with QRAP is governed not only by the numerical aperture but also by other parameters such as the diameter of pinholes in QRAP and the diameter of pinhole used for recording the point spread functions (Supplementary sections S2, S7 and S8). The photon budget calculations indicate that the indirect imaging method has lower light efficiency of about two orders than direct imaging systems (Supplementary section S4) for a typical imaging scenario and can be four orders while recording the point spread functions. One of the problems arising from the depth-wavelength reciprocity is that it is difficult to understand the source of a blur, whether it is due to a change in wavelength or depth. Therefore, the depth-wavelength reciprocity leads to depth-wavelength ambiguity i.e., a blur in a reconstruction can be considered as a superposed state made up of two equally likely events of change of wavelength or depth. The superposed state can be reduced to one of the two states by prior knowledge of the system. While recording the PSFs, there are three degrees of freedom (*u*, *v*, *λ*) but while observing an event there are only two degrees of freedom (*u*, *λ*) and so the probability of generating a change in wavelength by changing only *u* with *v* fixed is quite low. However, further studies are needed to eliminate this ambiguity. Furthermore, it is must be noted that the depth-wavelength reciprocity is valid only for single scattering and within the paraxial region of Fresnel approximation. Meta optics and machine learning modules can be incorporated into the technique in the future for precise engineering of wavefronts to improve the signal to noise ratio and resolution of the technique^39^.

In this study, an indirect imaging technique using chaotic waves have been proposed and demonstrated with advanced capabilities beyond what is possible with direct imagers. The capability to record a scene or event without a lens and decode that into multiple spatial and spectral channels will greatly benefit many research areas and industrial imaging applications. Some key areas are listed below: (a) Ultrafast imaging: Most of the ultrafast cameras available in the market are monochrome. It is almost impossible to record multispectral videos and at depths simultaneously at ultrafast timescales. If a conventional in-line holography technique was implemented, then multiple camera shots are needed with relative phase shifts which expands the time scale. The developed technique can revolutionize the area of ultrafast imaging by rendering hundreds or thousands of spatial and spectral channels from a single ultrafast video. This will enhance the understanding of the phenomenon occurring at ultrafast timescales. (b) Applicability range: The technique can be implemented using a quasi-random array of pinholes which is easy to create for any band of the electromagnetic spectrum and therefore the technique can be extended easily to other non-visible bands of the electromagnetic spectrum such as Terahertz and Infrared radiations for industrial applications. (c) Calibration time: The method requires lesser calibration time compared to the earlier studies^30,34^ as it is sufficient to record the point spread functions only at the axial and spectral boundaries involving four camera shots and all other point spread functions can be synthesized^40^. (d) Hyperspectral imaging and spectrometry: The technique is a natural spectrometer as it is possible to get the spectrum for every pixel of an image. (e) Fluorescence microscopy: The technique uses only incoherent illumination with source characteristics like that of fluorescence emission and so fluorescence microscopes can be built on the developed concept. (f) Experimental requirements: The technique is a lensless, intereferenceless, motionless, non-scanning, single camera shot, 5D imaging technique. Therefore, the technique is much simpler to implement without the cumbersome requirements of any other existing inline holography techniques. (g) Aberration: The technique has self-aberration correction capabilities due to the indirect mode of imaging. (h) Field of view: The technique possesses at least twice the field of view of any equivalent direct imaging system. (i) Cost: Above all, the technique requires only a few low-cost components such as a web camera, computer, a LED and a pinhole array (with holes tens-of-micrometers in diameter for most of the practical imaging tasks).

## Materials and methods

### Theory of multispectral and spatial imaging with a chaotic wave

First, we present the fundamental differences between incoherent imaging using a single pinhole or a lens and a RAP. Imaging an object using a pinhole or lens creates an image of the object on the image sensor. When imaging an object using a RAP, a random array of images is generated in the image plane. The recorded intensity pattern is the sum of the randomly located replica of the image of the object. This is true whether the object is a point object or a more complicated two-dimensional object. For a spatially incoherent illumination, an object can be considered as a collection of uncorrelated point objects. If *I*_*PSF*_ is the point spread function, then the object intensity pattern *I*_*O*_ of an object *O* can be expressed as a convolution of the object with the *I*_*PSF*_ i.e., $I_{{\text{O}}} = O \otimes I_{PSF}$, where ‘⊗’ is the 2D convolutional operator. Therefore, the image *O*’ of object *O* can be reconstructed by *I*_O_*I_*PSF*_, where ‘*’ is a 2D correlation operator. For a 3D object illuminated by many wavelengths, *I*_O_ can be considered as a sum of shifted and scaled *I*_PSF_s corresponding to different depths *z* and wavelengths *λ* expressed as $I_{{\text{O}}} = \mathop \sum \limits_{i,j}^{p,q} O\left( {\lambda_{i} ,z_{j} } \right) \otimes I_{PSF} \left( {\lambda_{i} ,z_{j} } \right)$, where *p* and *q* are the number of wavelengths and depths and *i* = 1,2,3…*p* and *j* = 1,2,3…*q*. If a library of $I_{PSF} \left( {\lambda_{i} ,z_{j} } \right)$ was pre-recorded for different values of *z* and *λ*, then *I*_O_ can be reconstructed into wavelength and depth specific images of the object. A detailed theoretical analysis is presented in the Supplementary Materials (Section S1). A pinhole camera is not highly sensitive to changes in axial distance or wavelength. Spectral changes are often not detected due to the larger spectral bandwidth of the focus of refractive lenses. In contrast, when an object is imaged using a RAP, unique random spatio-spectral intensity signatures are obtained for different values of depth and wavelength. The extracted multispectral and spatial channels can be computationally uniquely stained and combined to generate a multispectral 3D image.

**Figure 6:**
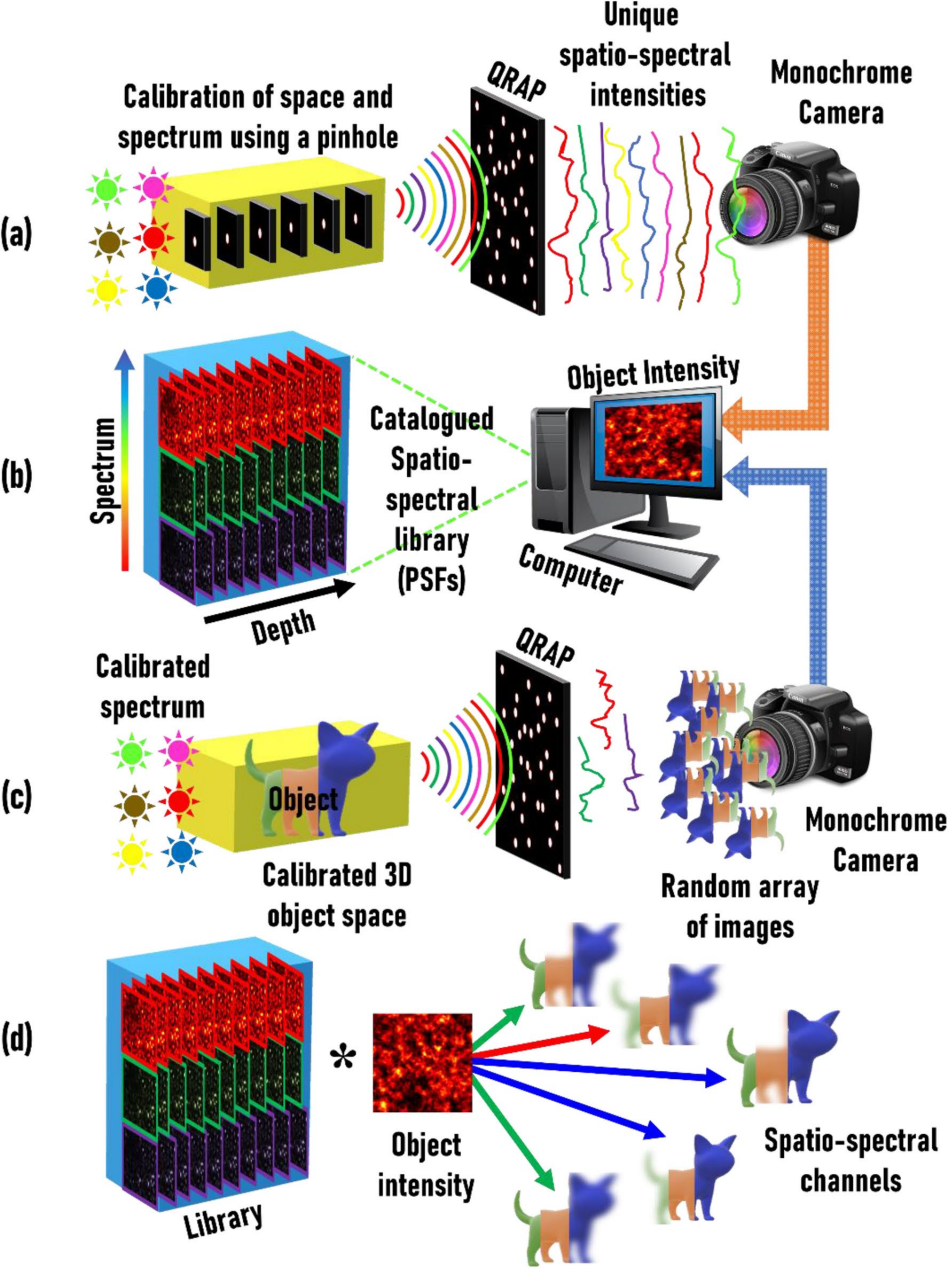
Optical configuration of imaging using a RAP mask. (**a**) Manual training process involving axially scanning a pinhole and changing the illumination wavelength at every location and recording the spatio-spectral library using a monochrome camera for a QRAP. (**b**) The recorded library is catalogued for different wavelength and depth parameters. (**c**) A thick multispectral object is placed within the calibrated space and a single camera shot is recorded for the object under identical experimental conditions. (**d**) The image of the object in five dimensions is reconstructed by a computational correlation of the object intensity pattern with the library, ‘$*$’ is a 2D correlator.

A schematic of the multispectral and spatial imaging system, optical and computational processes are presented in Fig. 6. Light from an incoherent source illuminates a pinhole and the diffracted light is modulated by a quasi-random array of pinholes (QRAP) and the intensity pattern is recorded by a monochrome camera. A library of spatio-spectral intensity signatures is recorded, catalogued and stored as a library function *I*_*PSF*_(*z*,*λ*). The above process is done only once. An object is placed within the axial boundaries of the library and illuminated by wavelengths within the spectral limits of the library and the object intensity pattern *I*_O_ is recorded. The *I*_O_ is recorded under identical conditions and distances as *I*_*PSF*_(*z*,*λ*). The spatially and spectrally resolved image is reconstructed using a cross-correlation between the *I*_*O*_ and *I*_*PSF*_.

The indirect imaging technique using QRAP can be considered as a pattern recognition problem. In the first step, the point spread functions which are the unique spatial and spectral signatures are recorded. These signatures are the reference patterns catalogued in wavelength and depth. The intensity pattern generated for any complicated object is the summation of the shifted and scaled reference patterns. Therefore, when the object intensity pattern is cross-correlated with the reference patterns, a peak is obtained wherever there is a match. In this way, the spatial and spectral composition of the object can be known without any external validation.

One of the characters of the chaotic waves studied above is the scattering ratio. Smaller is the diameter of the pinholes, larger is the scattering angles of the chaotic wave and higher is the lateral, axial and spectral resolving powers. With higher values of scattering angles, it is possible to reach the resolution limits of direct imaging. A secondary constraint on imaging is the size of the pinhole used to record the point spread function libraries. The Rayleigh resolution limits are applicable only when the sampling point object is a real point object. However, in the experiment, the pinholes have diameters of few tens of micrometres and therefore imposes limits on the resolutions resulting in a lower value than the theoretical ones. The reconstructed image, $O^{\prime} = O \otimes I_{PSF} *I_{PSF}$ which reduces to, $O^{\prime} = O \otimes \wedge$, where Λ is a delta-like function obtained by the autocorrelation of *I*_*PSF*_ which cannot be below the size of the smallest speckle created by the self-interference of the chaotic waves. In other words, the spatial frequency at which the object gets sampled in an imaging system depends upon the autocorrelation function which is dependent upon the size of the pinholes in the QRAP and the pinhole used for recording the point spread intensity distributions.

### Complex field simulations

The complex field simulations were carried out using scalar diffraction formulations with Fresnel approximation. All objects were assumed to be made up of many number of independent point sources emitting light with no mutual coherence resulting in intensity addition in the sensor. The simulation studies and calculations were processed by cross-correlating them with the spatio-spectral library using the most recently developed non-linear filter and the optimal reconstruction parameters were obtained. In addition to the non-linear filter, a low-pass filter, median filter and a cross-correlation at the spectrum domain were carried out in sequence to obtain the reconstruction with the highest signal to noise ratio. The beam propagation studies and calculations were carried out using MATLAB and Octave.

### Optimization of the pinhole array

A random array of pinholes was synthesized using two random number generators. The synthesis is iterated 1,000 times and every time, the pinhole array was implemented in the complex field simulations and the background noise was estimated. The best arrangement of pinholes with the highest SNR was selected for the second round of optimization. In the second step, a search algorithm was implemented by shifting the location of every pinhole around its’ initial location by a predefined step size and the pinhole locations for the highest SNR was selected by simulating the respective complex fields every time. The second optimization involved about 10,000 iterations. The optimization was carried out in MATLAB.

### Fabrication of the pinhole array

The CAD design for the pinhole array was made from the image files from MATLAB and converted into DXF using the trial version of the LinkCAD software. The final mask design was transferred to the resist coated chromium mask plates using Intelligent micropatterning SF100 XPRESS. The pinholes were transferred to the chromium layer using a chrome etchant. The size of the QRAP was 8 mm × 8 mm, the diameter of the pinholes was 80 μm after fabrication and consisted of a total of approximately 2,000 pinholes.

### Experiments, data acquisition and reconstruction

The experiment was carried out using three light sources: Thorlabs (M617L3, *λ*_*c*_ = 617 nm, FWHM = 18 nm), (M530L3, *λ*_*c*_ = 530 nm, FWHM = 33 nm) and a white light source (Fiber-Lite, DC-950, Dolan-Jenner Industries) with a 488 nm chromatic filter. For reflection mode illumination, the white light source with flexible fibre output was used with a filter in the image sensor. The USAF and NBS resolution targets used for the experiments are R1DS1N (Thorlabs) and R2L2S1N (Thorlabs) respectively. The intensity patterns were recorded using Thorlabs camera (DCU223M, 1,024 × 768 pixels, pixel size = 4.65 μm) and the images were grabbed using Thorlabs software. The reconstruction was carried out in MATLAB and Octave.

## Supplementary information

Supplementary information.
