# Supplementary material for: Single shot multispectral multidimensional imaging using chaotic waves
Source: Sci Rep. 2020 Aug 17;10:13902. doi: 10.1038/s41598-020-70849-7 (PMC7431426; doi:10.1038/s41598-020-70849-7)
Supplement: Supplementary file 1 — Supplementary information. [file 41598_2020_70849_MOESM1_ESM.pdf]

Supplementary materials for

Single Shot Multispectral Multidimensional Imaging using Chaotic Waves

Authors

Vijayakumar Anand,[1,*] Soon Hock Ng,[1] Jovan Maksimovic,[1] Denver Linklater,[2] Tomas Katkus,[1] Elena P Ivanova,[2] Saulius Juodkazis[1,3,4]

Affiliations

[1]Center for Micro-Photonics, Faculty of Science, Engineering and Technology, Swinburne University of Technology, Hawthorn, VIC 3122, Australia.

[2]Department of Physics, RMIT, GPO Box 2476, Melbourne VIC 3001, Australia

[3]Melbourne Centre for Nanofabrication, ANFF, 151 Wellington Road, Clayton, VIC 3168, Australia.

[4]Tokyo Tech World Research Hub Initiative (WRHI), School of Materials and Chemical Technology, Tokyo Institute of Technology, 2-12-1, Ookayama, Meguro-ku, Tokyo 152-8550, Japan.

**Corresponding author:* vanand@swin.edu.au

The PDF file includes

Supplementary text
Section S1. Theoretical Analysis
Section S2. Lateral resolution of an imaging system using chaotic waves
Section S3. Imaging with a wide field of view using chaotic waves
Section S4. Photon budget
Section S5. Optimization Procedure for RAP
Section S6. Non-linear correlation and adaptability tests
Section S7. Axial resolution of an imaging system using chaotic waves
Section S8. Spectral resolution of an imaging system using chaotic waves
Section S9. Depth-Wavelength relationships
Section S10. Computational modules for automation
Section S11. Octave code for automation
References

Supplementary Text

Section S1. Theoretical Analysis

The optical configuration is given in **Fig. S1**. The theoretical analysis is from the object plane (o plane) to the sensor plane (s plane) [1]. Light from an incoherent point located at $(\overline{r_o}, u)$ with an amplitude of $\sqrt{I_o}$ reaches the plane of the mask with a complex amplitude given by $C_1\sqrt{I_o}L(\overline{r_o}/u)Q(1/u)$, where C_1 is a complex constant, $\overline{r_o} = (x_o, y_o)$, $L(\overline{o}/u) = \exp[j2\pi(o_x x + o_y y)/(\lambda u)]$ and $Q(1/u) = \exp[j\pi(x^2 + y^2)/(\lambda u)]$ are the linear and quadratic phase factors. The mask located at the mth plane consisting of a random array of N pinholes can be expressed as $\sum_{i=1}^{N}\delta(\overline{r} - \overline{r}_{i,m})\otimes circ(R)$, where $\delta(\overline{r} - \overline{r}_{i,m})$ is a Delta function, R is the radius of the pinhole and '⊗' is a two dimensional convolutional operator, $\overline{r_m} = (x_m, y_m)$, $\overline{r_i} = (x_i, y_i)$ and $x_i, y_i \in \{C_2 \cdot (U[0,1])\}$, U is a uniform random variable distributed on [0, 1]. The above convolution operation creates circles in the locations of the Delta functions. The complex amplitude after the mask is given as $C_1\sqrt{I_o}L(\overline{r_o}/u)Q(1/u)\left[\sum_{i=1}^{N}\delta(\overline{r} - \overline{r}_{i,m})\otimes circ(R)\right]$.

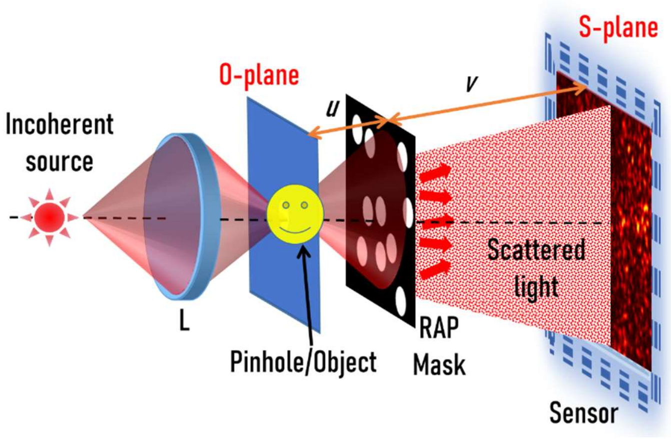

Fig. S1 Optical configuration of indirect imaging using a RAP mask. Light from an incoherent source illuminates the object in critical illumination configuration. The light diffracted by the object is modulated by the RAP Mask and the scattered light is recorded by the image sensor. The variables u and v are the distances between the object and RAP mask and RAP mask and the image sensor respectively.

The intensity pattern recorded by the image sensor for a single point is given as

$$I_{PSF}(\overline{r_s}; \overline{r_o}, u) = \left|C_1\sqrt{I_o}L\left(\frac{\overline{r_o}}{u}\right)Q\left(\frac{1}{u}\right)\left[\sum_{i=1}^{N}\delta(\overline{r} - \overline{r}_{i,m})\otimes circ(R)\right]\otimes Q\left(\frac{1}{v}\right)\right|^2. \quad \textbf{(S.1)}$$

The above equation can be expressed as

$$I_{PSF}(\overline{r_s}; \overline{r_o}, u) = I_{PSF}\left(\overline{r_s} - \frac{v}{u}\overline{r_o}; 0, u\right). \quad \textbf{(S.2)}$$

A two-dimensional object located in the object plane o when illuminated by a spatially incoherent light can be considered as a collection of M point objects given by

$$o(\overline{r_o}) = \sum_{i=1}^{M} a_i\,\delta(r - r_i). \quad \textbf{(S.3)}$$

Due to the lack of spatial coherence, the object intensity pattern obtained in the *s* plane with the same mask is the summation of the shifted and scaled point spread intensity functions given as

$$I_o(\overline{r_s}, u) = \sum_{i=1}^{M} a_i \, I_{PSF}\left(\overline{r_s} - \frac{v}{u}\overline{r_{o,i}}; 0, u\right) \quad \textbf{(S.4)}$$

The image of the object is reconstructed by a cross-correlation between I_o and I_{PSF}

$$I_R(\overline{r_R}) = \iint \sum_{i=1}^{M} a_i \, I_{PSF}\left(\overline{r_s} - \frac{v}{u}\overline{r_{o,i}}; 0, u\right) I_{PSF}^{*}(\overline{r_s} - \overline{r_R}; 0, u)$$

$$= \iint \sum_{i=1}^{M} a_i \, \Lambda I_{PSF}\left(\overline{r_s} - \frac{v}{u}\overline{r_{o,i}}; 0, u\right) \approx o\left(\frac{\overline{r_s}u}{v}\right), \quad \textbf{(S.5)}$$

where the transverse magnification M_T=(v/u) and Λ is a delta-like function with a maximum at the origin and negligible values in places other than the origin. The above analysis shows that it is possible to reconstruct the object information by a cross correlation between the object intensity pattern and the point spread function. For an object consisting of multiple planes and illuminated by different wavelengths, the object intensity pattern is the summation of the object intensity patterns at different planes and different wavelengths $I_o(\overline{r_s}) = \sum_{i,j=1}^{p,q} I_o(\overline{r_s}, u_i, \lambda_j)$ which can be reconstructed at different wavelengths and depth using $I_{PSF}(\overline{r_s}, u_i, \lambda_j)$, where p and q are the number of wavelengths and depths, i=1,2,3…p and j=1,2,3…q.

Section S2. Lateral resolution of an imaging system using chaotic waves

A schematic of a basic multispectral sequential direct imaging system is shown in **Fig. S2**. In order to have a reliable comparison, we consider the direct imaging with a single lens in place of the pinhole array but with a wavelength tunable light source, an object and a monochrome sensor. The above method is also equivalent to illuminating an object with a white light source and using chromatic filters with different central wavelength at the image sensor. A tunable light source is used to illuminate a colour object. The light diffracted by the object is imaged by a monochrome image sensor. By changing the wavelength of the light source in increments, the images of the object corresponding to different wavelengths can be recorded sequentially in time.

The lateral resolution of RAP is calculated by simulation of the random distribution for u = 10 cm, v = 10 cm, D = 2 mm, λ = 600 nm and n = 100, where n is the number of pinholes, for pinhole diameters varying from 10 μm to 50 μm. The lateral resolution of a direct imaging system with the above configuration with a lens of focal length f = 5 cm instead of the pinhole array is given as Δ_l = 1.22$\lambda u/D$ ~ 37 μm. The size of the smallest speckle that can be formed with the RAP cannot be smaller than the lateral resolution limit of direct imaging as the mean diameter of the speckle is 1.22$\lambda u/D$ [2, 3]. As seen from the relation, the speckle size appears to be dependent only on the numerical aperture (NA) and the wavelength. However, the above expression is only a limiting value and the speckle size depends upon the scattering degree of the scattering function of RAP. In the indirect imaging method with RAP, the lateral resolution is given by the autocorrelation of the random intensity distribution. Therefore, the smallest spot that could be obtained with RAP is ~2.44$\lambda u/D$. However, with different filters, the smallest speckle size could be tuned to match with 1.22$\lambda u/D$ [4].

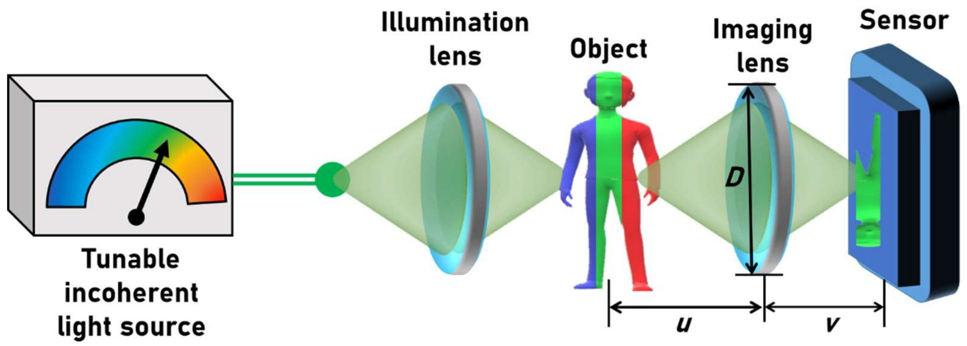

Fig. S2 Optical configuration of sequential multispectral direct imaging system.

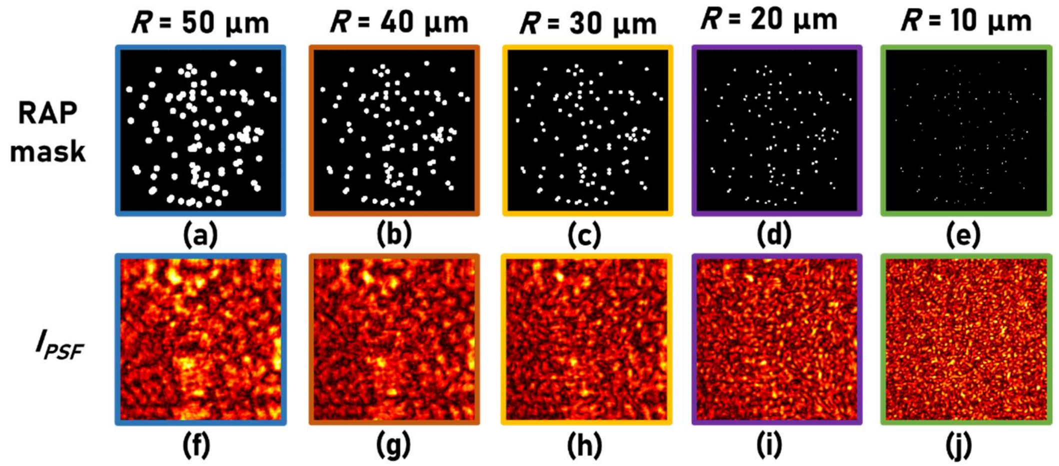

Fig. S3 RAP mask and random intensity distributions. Images of the RAP mask for (a) R = 50 μm, (b) R = 40 μm, (c) R = 30 μm, (d) R = 20 μm and (e) R = 10 μm. Random intensity distributions for (f) R = 50 μm, (g) R = 40 μm, (h) R = 30 μm, (i) R = 20 μm and (j) R = 10 μm.

The images of the RAP for R = 50 μm to 10 μm in steps of 10 μm are shown in **Figs. S3a-S3e** respectively. The images of the I_{PSF}s for R = 50 μm to 10 μm are shown in **Figs. S3f – S3j** respectively. From the figures, it is seen that the speckle size decreases and visibility increases with a decrease in the diameter of the pinhole and reaches the limiting value $\Delta_l = 1.22\lambda u/D \sim$ 37 μm for this configuration. The plot of the autocorrelation function with a phase only filter for R = 10 μm to 50 μm in steps of 5 μm is shown in **Fig. S4** [5]. From the plot, it is seen that the resolving power of the system improves with a decrease in the size of the pinholes. The offset seen in indirect imaging with RAP is because correlation between two positive functions results in a background. The FWHM of the autocorrelation function is plotted as a function of the diameter of the pinholes in **Fig. S5** and the data is analysed to obtain a semi-empirical relationship between the diameter of the pinhole and the diameter of the autocorrelation function including the factors λ, u, and D. The FWHM of airy disk for direct imaging is $\sim 0.52\lambda u/D$= 16 μm. The variation of the FWHM is linear with respect to the variation in the diameter of the pinholes. The following approximate semi-empirical relationship has been established between the radius of pinhole and the FWHM as $\Delta_{l\text{-}FWHM}(R) \sim 0.52\lambda u/D + K\times(R\text{-}5)$ for $R \geq 5$ μm, and the proportionality factor K=3/4 is the slope of the variation of the FWHM of the autocorrelation function with respect to the radius of the pinhole.

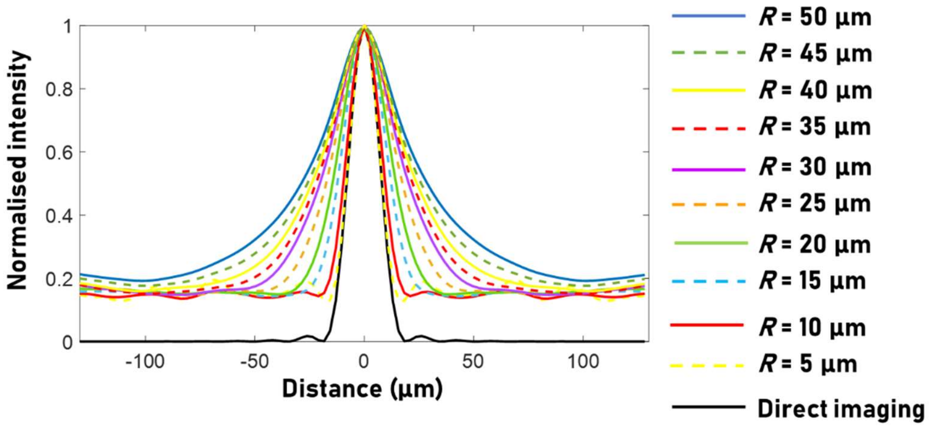

Fig. S4 Plot of the autocorrelation function. The radius of the pinhole was varied from 10 µm to 50 µm in steps of 5 µm and the normalised autocorrelation function is plotted as a function of lateral distances.

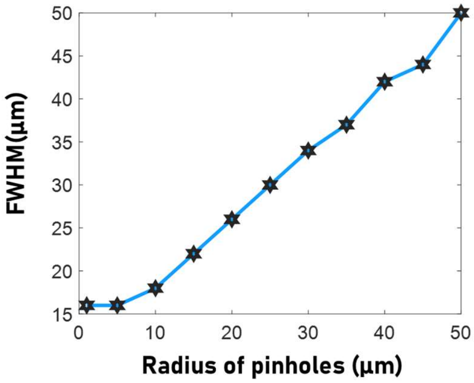

Fig. S5 FWHM of autocorrelation function vs Radius of pinholes. The radius *R* of the pinholes was varied from 5 µm to 50 µm in steps of 5 µm and the corresponding FWHM of autocorrelation functions were calculated and plotted.

Section S3. Imaging with a wide field of view using chaotic waves

The field of view (FOV) of an imaging system is limited by the magnification of the system and the size of the sensor. From **Fig. S6**, the FOV of the imaging system is given as ~(su/v). In a direct imaging system, when an object is imaged, the points of the object which lie beyond the FOV are not recorded indicated by the red points in both the object and the image in **Fig. S6(a)**. Imaging using a RAP, generates an intensity pattern which is the superposition of the random object images, an original perspective is shown in **Fig. S6(b)**. This does not give any additional information about the FOV of the imaging system. However, an alternative perspective of the same system is presented in **Fig. S6(c)**. As per this view, any off-axis object point will generate a shifted spatio-spectral signature. Therefore, every object point is converted not into an image point like the direct imaging system but into a spatio-spectral signature which is much larger in area than the sensor.

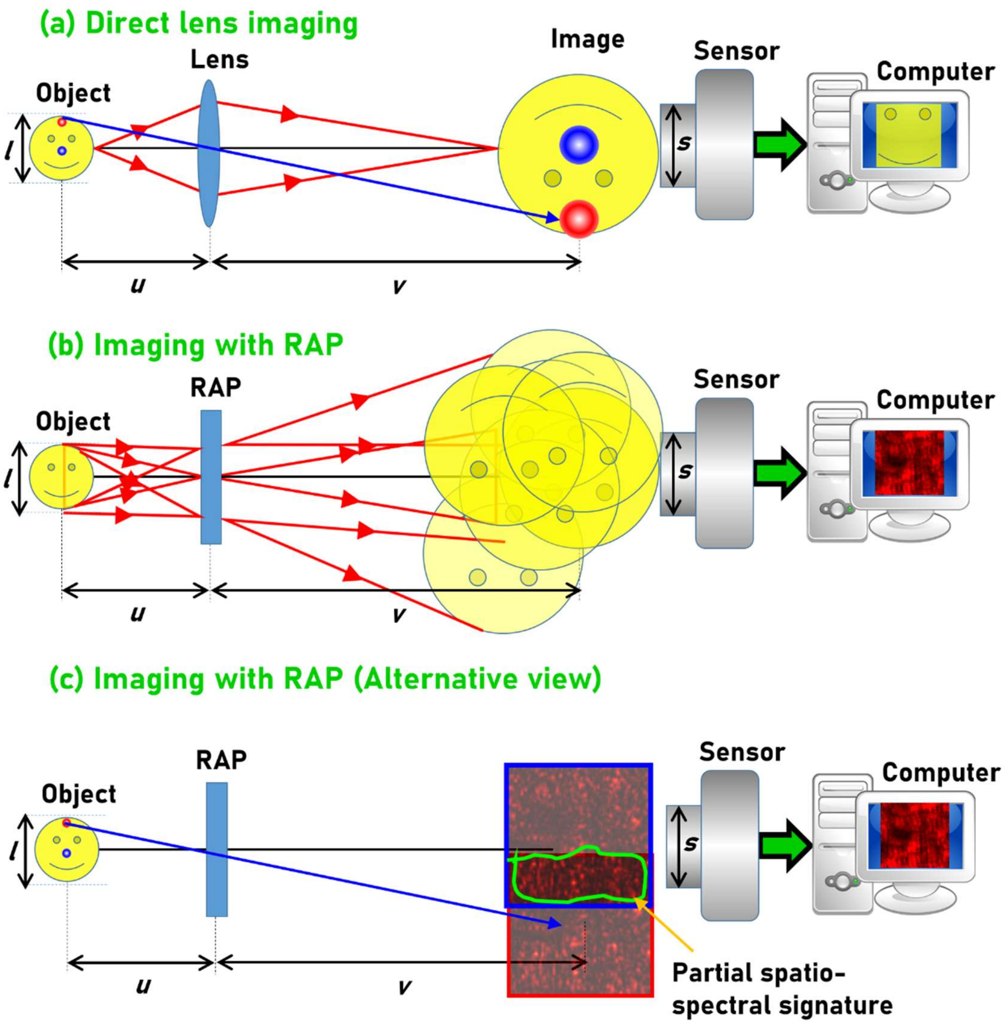

Fig. S6 Optical configuration of imaging, (**a**) lens, (**b**) RAP. **(c)** An alternative view of imaging using RAP.

Therefore, even when the object point lies beyond the FOV, i.e., the centre of the spatio-spectral signature is beyond the area of the sensor, there is always some partial spatio-spectral signature that is incident on the image sensor. If the library of spatio-spectral signatures has been acquired synthetically over a larger area only once, then any object imaged using RAP can be imaged with a high FOV compared to the direct imaging system. Even without such training, from the figures, it is clear that in order to move an object point beyond the FOV of the direct imaging system the object point must be shifted by $\pm su/2v$ from the centre. On the other hand, a shift of at least $\pm su/v$ from the centre is needed in the case of imaging using RAP in order to not have any partial spatio-spectral signature present in the image sensor. For u = 10 cm and v = 10 cm, S = 4 mm, the limits are ± 2 mm and ± 4 mm for direct and indirect imaging respectively.

Section S4. Photon budget

The photon budget is compared between direct imaging and the proposed indirect imaging with chaotic waves using **Fig. S7**. A circular disk of object with a diameter of d_1 illuminated critically by an incoherent light source is considered in the object plane. The disk has a uniform intensity distribution and I_o is the intensity at any point within the disk, the optical power at the

plane-A is $P_o = I_o \pi d_1^2/4$. The light diffracted from the disc has a Bessel intensity distribution with a central maximum of diameter $2.44\lambda u/d_1$. Within the central maximum, about 84% of the optical power is distributed. The power reaching the lens or the RAP within an area with a diameter D at plane-B is $k_{AB}P_o$, where k_{AB} is the power ratio between plane-B and plane-A. The power reaching plane-B is identical for both imaging systems. In the case of direct imaging using a lens, there is loss of optical power due to Fresnel reflection R_f at the air-glass and glass-air interfaces. The fraction of light entering the lens is $k_{AB}P_o(1-R_f)$. Therefore, the light exiting the lens at plane-C has an optical power given by $k_{AB}P_o(1-R_f)^2$. The image of the disc is formed at the plane-D in the sensor of area $S\times S$. The diameter of the image of the disc is given as $\sim\sqrt{(2.44\lambda v/D)^2 + {d_1}^2(v/u)^2}$. The first term in the expression is the diameter of the airy disk and the second term is the image of the disk. In order to have a better approximation, the root squared sum approach is used here. The intensity is therefore given as ${I_s}^D \sim \left\{k_{AB}P_o\left(1-R_f\right)^2\right\}/\left[\frac{\pi}{4}\left\{(2.44\lambda v/D)^2 + {d_1}^2(v/u)^2\right\}\right]$.

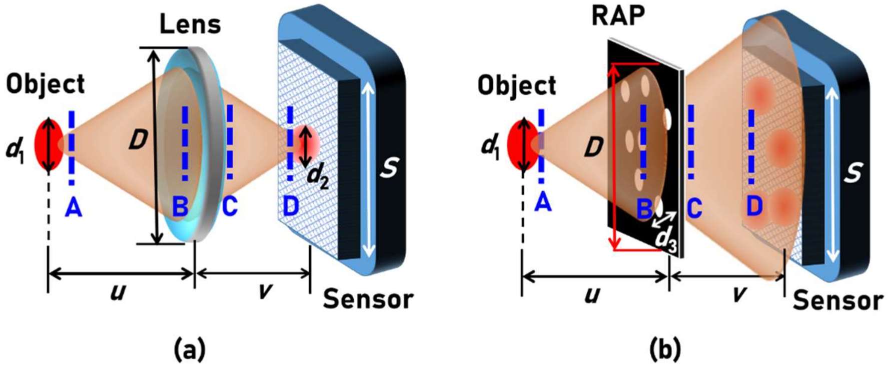

Fig. S7 Optical configuration of (a) direct imaging and (b) indirect imaging using chaotic waves.

For the indirect imaging system using RAP, the analysis begins from plane-B where the optical power is $k_{AB}P_o$. The RAP consists of n pinholes each with a diameter of d_3. Hence, the total area of pinholes to the overlap area of the RAP mask with the diffracted light is given as $k_{BC} = n{d_3}^2/D^2$. It is assumed that $d_1 >> d_3$, therefore, each and every pinhole in RAP generates an image of the disc and the summation of these shifted images is obtained in the sensor. The maximum diameter of the random intensity distribution is given as $\sim\left[D\{1+(v/u)\} + \sqrt{(vd_1/u)^2 + (2.44\lambda v/d_3)^2}\right]$. It is assumed that the random intensity distribution follows a uniform random variable (2D locations of pinholes) with a uniform probability distribution function and so the intensity distribution can be considered uniform. However, there are localization effects due to the self-interference phenomenon resulting in a redistribution of light from out-of-phase wavelets to in-phase wavelets. The fraction of light available within the sensor area is given as $\sim 4S^2/\pi\left[D\{1+(v/u)\} + \sqrt{(vd_1/u)^2 + (2.44\lambda v/d_3)^2}\right]^2$ for $S < \left[D\{1+(v/u)\} + \sqrt{(vd_1/u)^2 + (2.44\lambda v/d_3)^2}\right]$. At plane-$D$, the intensity is given as ${I_s}^I \sim 4k_{AB}k_{BC}P_o/\pi\left[D\{1+(v/u)\} + \sqrt{(vd_1/u)^2 + (2.44\lambda v/d_3)^2}\right]^2$. If the pixel size of the sensor is Δ then the optical power in each pixel for direct imaging system is

${P_s}^D \sim 4\Delta^2 \left\{k_{AB}P_o\left(1-R_f\right)^2\right\}/\left[\pi\left\{(2.44\lambda v/D)^2 + {d_1}^2(v/u)^2\right\}\right]$ and indirect system is ${P_s}^I \sim 4\Delta^2 k_{AB}k_{BC}P_o/\pi\left[D\{1+(v/u)\}+\sqrt{(vd_1/u)^2+(2.44\lambda v/d_3)^2}\right]^2$. The number of photons incident on the pixel of sensor per second is ${N_s}^D \sim 4\lambda\Delta^2 \left\{k_{AB}P_o\left(1-R_f\right)^2\right\}/\left[hc\pi\left\{(2.44\lambda v/D)^2 + {d_1}^2(v/u)^2\right\}\right]$ for the direct imaging system and ${N_s}^I \sim 4\lambda\Delta^2 k_{AB}k_{BC}P_o/\pi hc\left[D\{1+(v/u)\}+\sqrt{(vd_1/u)^2+(2.44\lambda v/d_3)^2}\right]^2$ for the indirect imaging system, where h is the Planck's constant and c is the velocity of light in vacuum.

A case study is considered and the number of photons is compared between direct imaging system and the indirect imaging system with a RAP. For u = 10 cm and v = 10 cm, d_1 = 1 mm, d_3 = 0.1 mm, λ = 600 nm, n = 100, D = 2 mm, R_f = 0.04 (n_1=1.5 and n_2 = 1), P_o = 100 mW and Δ = 4 μm. The diameter of the airy disk at plane-B is ~ 146 μm which is much smaller than D and so k_{AB} ~ 1. k_{BC} = 0.25. The number of photons per second incident on a pixel of the camera for the direct imaging system is ~ 6×10^{12} photons. The number of photons per second incident on a pixel of the camera for the indirect imaging system is ~ 5×10^{10} photons. The ratio of the number of photons per pixel per second for direct imaging to indirect imaging is 120. However, for a point object such as a pinhole (20 μm) as used in the experiment, the ratio is ~20000. However, the above ratio is not surprising as it matches with the ratio between typical holography systems and direct imaging methods.

Section S5. Optimization Procedure for RAP

A random array of pinholes is synthesized using two uncorrelated random variables $x_i, y_i \in \{C_2 \cdot (U[0,1])\}$, where U is a uniform random variable distributed on [0, 1], while x_i, y_i are uniform random variables distributed on [0, C_2]. To reduce the background noise, quasi-random variables u_i, v_i were synthesized such that the mask consisting of the pinholes at the new locations have an improved SNR. The SNR is defined as Signal/(Average background noise) which reduces to 1/(Average background noise) upon normalizing the reconstructed intensity pattern. The two-step optimization procedure is shown in **Fig. S8**. The random variables x_i, y_i are iterated over N times and the random variables u_i, v_i corresponding to maximum SNR is selected and given as input to the second stage optimization procedure. In the next stage, the location of the pinholes is shifted along the X and Y directions with the limits $-L \leq \Delta u_i \leq L$ and $-L \leq \Delta v_i \leq L$ both in steps of Δ and the SNR is calculated at every step and the quasi-random variables ${u_i}'$, ${v_i}'$ corresponding to the maximum SNR is determined. The second optimization, therefore, runs over $2L/\Delta$ iterations for every location along X or Y direction. Therefore, the total number of iterations in the second optimization is $4nL/\Delta$.

A specific design condition of object distance (u = 10 cm) and image distance (v = 10 cm), wavelength (λ = 617 nm), pinhole diameter (d = 80 μm), mask diameter (D = 8 mm) and number of pinholes (n = 2000) is considered. During every iteration, the light from a point object is propagated by 10 cm and modulated by the RAP mask and the modulated light is propagated by another distance of 10 cm. The diameter of the pinholes was selected to be 80 μm and the wavelength was selected to be 617 nm. The intensity distribution at 10 cm from the quasi RAP (QRAP) mask is autocorrelated using a phase-only filter [5] as $I_{PSF} * {I_{PSF}}'$, where $I'_{PSF} = \left|\mathcal{F}^{-1}\left\{exp\left[i\, arg(I_{PSF})\right]\right\}\right|$ and the SNR was calculated as 1/(Average background noise) after normalizing the maximum intensity value of the autocorrelation. The plot of the SNR with the number of iterations is shown in **Fig. S9**. The image of the RAP masks and the reconstructed images with the phase-only filter for an object "NANO LAB" using the masks with the

minimum SNR, maximum SNR after step – 1 and maximum SNR after step – 2 respectively are shown in **Fig. S10**. The improvement in the SNR is clearly visible with an improvement of 64%. The final mask design was transferred to a chromium coated mask plate using Intelligent micropatterning SF100 XPRESS. The size of the QRAP was 8 mm and the diameter of the pinholes was 80 µm after fabrication.

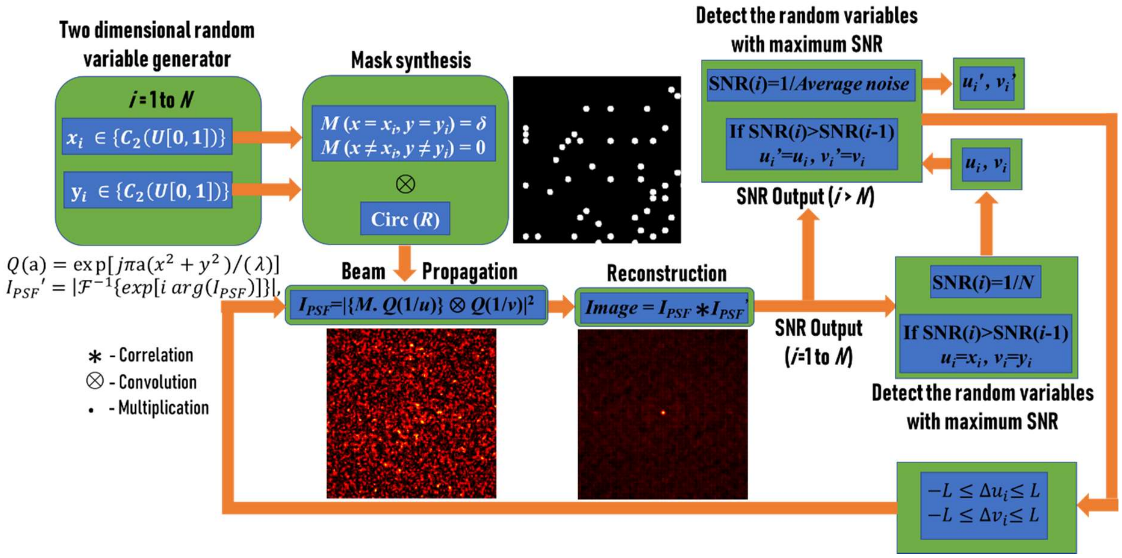

Fig. S8 Optimization procedure for designing QRAP. Two-steps optimization procedure to improve the SNR of reconstruction. In the first step, two random number generators are used to synthesize RAP masks 1000 times and the RAP mask with the highest SNR is given as input to the next step. In this step, the location of every pinhole is shifted, and the optimal locations are determined iteratively.

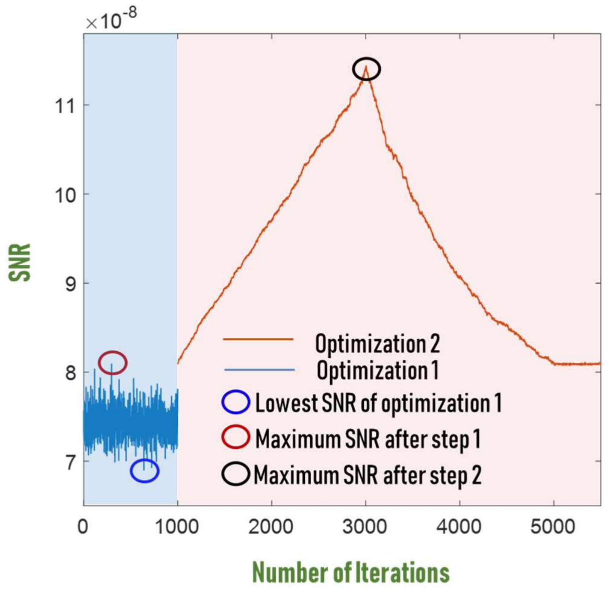

Figure S9. **Plot of the SNR as a function of the number of iterations.** The blue shaded region indicates the iterations using optimization step – 1 and the orange shaded region indicates the iterations using the optimization step – 2. In the optimization process – 1, the random array generator was iterated 1000 times and the RAP profiles with lowest and highest SNR are

identified. The RAP profile with the highest SNR was selected for the second optimization where the location of every pinhole was shifted by ± 5 pixels and the profile with the highest SNR was identified. An overall SNR enhancement of 64% was achieved in comparison to the lowest SNR of optimization 1.

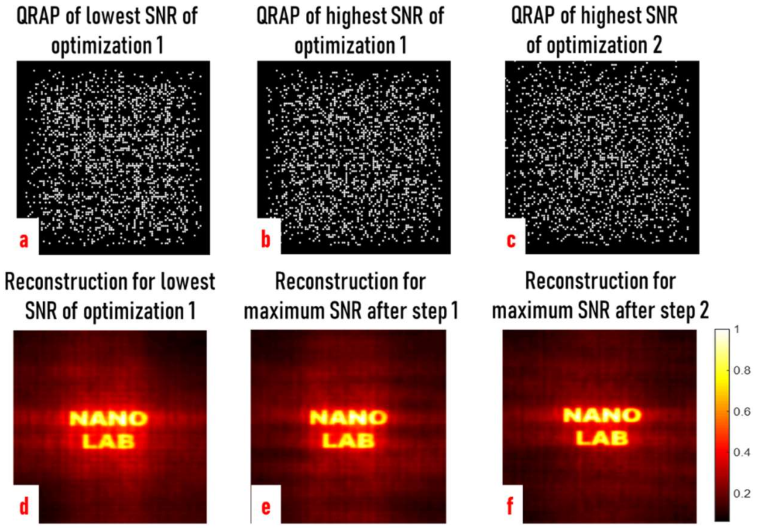

Fig. S10 Computational reconstruction results. QRAP of (**a**) lowest SNR of optimization 1, (**b**) highest SNR of optimization 1 and (**c**) highest SNR of optimization 2. Reconstruction result of a test object using QRAP of (**d**) lowest SNR of optimization 1, (**e**) highest SNR of optimization 1 and (**f**) highest SNR of optimization 2.

Section S6. Non-linear correlation and adaptability tests

A direct cross-correlation between an object intensity distribution and a point spread intensity distribution results in substantial background noise. The background noise is partially suppressed by optimizing the location of the pinholes. However, the background noise arising from the unipolar nature of the recorded intensity distributions is still present and will generate background noise during image reconstruction. In other words, a cross-correlation between two positive functions produces an offset or background noise. A direct method to solve this problem is to create a bipolar intensity pattern by recording two intensity patterns from two statistically different chaotic waves from the object and subtracting one from the other [1]. The above procedure demands two camera recordings for every object and requires either two QRAPs or requires rotating the QRAP to record a second intensity pattern. This decreases the temporal resolution of imaging. In non-linear correlation, the magnitudes of the spectrum of the two correlation functions are modified with respect to one another to create an effect equivalent to that of correlating two bipolar intensity distributions.

In the non-linear correlation, the object reconstruction can be expressed as

$$I_R = \left|\mathcal{F}^{-1}\left\{\left|\tilde{I}_{PSF}\right|^{\alpha} exp[i\, arg(\tilde{I}_{PSF})]\left|\tilde{I}_O{}'\right|^{\beta} exp[-i\, arg(\tilde{I}_O{}')]\right\}\right|, \quad \textbf{(S. 6)}$$

where the values of α and β are tuned between -1 to +1 until a case with minimum entropy is obtained. The entropy is expressed as $S(\alpha,\beta) = -\sum\sum\phi(m,n)log[\phi(m,n)]$, where $\phi(m,n) = |C(m,n)|/\sum_M\sum_N|C(m,n)|$, $C(m,n)$ is the correlation distribution, and (m,n) are the indexes of the correlation matrix.

An LED Thorlabs (M617L3, λ_c = 617 nm, FWHM = 18 nm) critically illuminated an object: United States Air Force (USAF) resolution target (Group – 2, Element – 6, 7.13 *lp/mm*). A pinhole with a diameter of 100 μm was used for sampling the system. The point object and object intensity distributions were recorded by an image sensor (Thorlabs DCU223M, 1024 x 768 pixels, pixel size = 4.65 μm) as shown in **Figs. S11(A)** and **S11(B)**. The distance between the object and the QRAP was 10 cm and the distance between the QRAP and the image sensor was 10 cm.

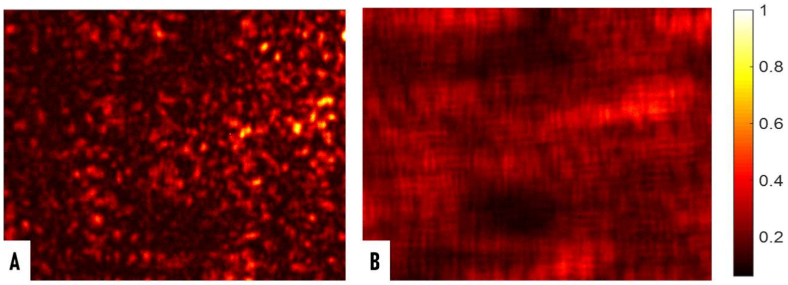

Fig. S11 Recorded intensity distributions. (A) Point spread intensity and (B) object intensity distributions.

The values of α and β were varied between -1 and 1 in steps of 0.2 and the entropy was calculated for every value of α and β. Since this is a test run, this is carried out only for **Figs. S11(A)** and **S11(B)**. The reconstructed images for all the values of α and β are shown in **Fig. S12**. The reconstructed images for matched filter (α = 1 and β = 1), phase-only filter (α = 0 and β = 1) and inverse filter (α = -1 and β = 1) are indicated using red, green and blue squares respectively. The optimal filter (α= 0 and β = 0.6) with entropy value 1 is indicated using a yellow square. The optimal filter is used for further reconstructions.

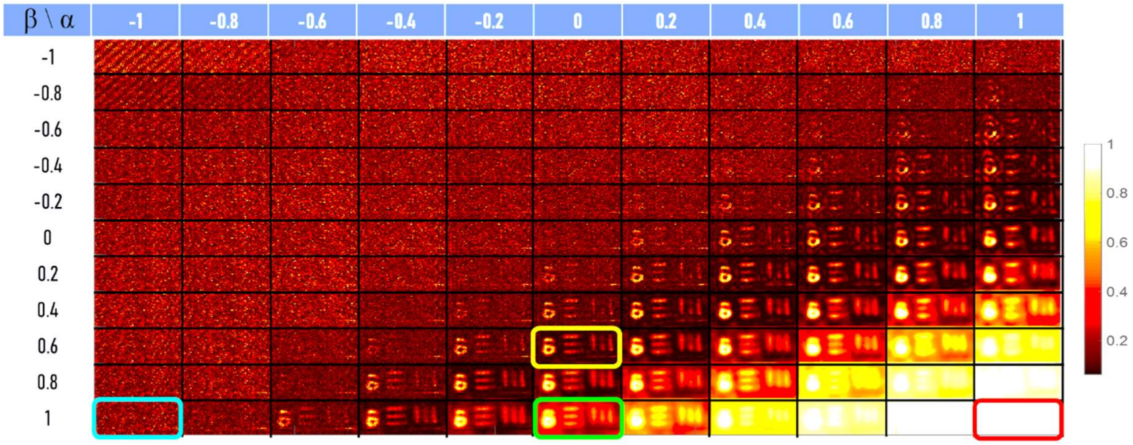

Fig. S12 Reconstruction results of the non-linear filter. α and β were varied between -1 and 1 in steps of 0.2. The optimal filter for entropy value 1 was found to occur at (α = 0 and β = 0.6). The results of the matched filter, phase-only filter and inverse filter are indicated by red, green and blue squares respectively.

The adaptability of the imaging technique with the non-linear correlator is evaluated next. Imaging using QRAP was repeated under a few extreme conditions and the performances were studied. In the first case, a part of a curved refractive element (a broken beaker) was introduced in between the object and the RAP as shown in **Fig. S13(A)**. The images of the point spread intensity and object intensity patterns are shown in **Figs. S13(B)** and **S13(C)** respectively. The

reconstructed image is shown in **Fig. S13(D)**. In the second case, a RAP was created by creating multiple holes on a black insulation tape. Then the insulation tape was then attached to a curved surface (same broken beaker) and was used for imaging as shown in **Fig. S13(E)**. The images of the point spread intensity, object intensity distributions and the reconstructed image are shown in **Figs. S13(F), S13(G) and S13(H)** respectively. From **Figs. S13(F) and S13(G)**, the distortions due to the curved surface of the beaker is visible. However, the image of the object was recovered with some additional noise. The above two cases were selected to demonstrate the level of flexibility available with the proposed technique.

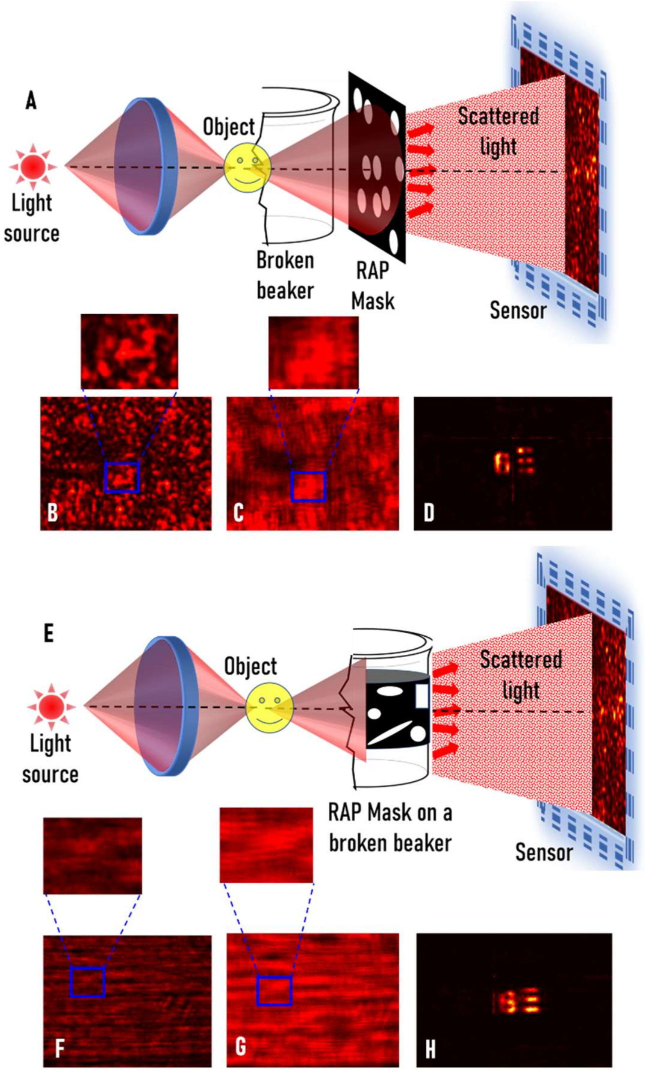

Fig. S13 Imaging under different conditions. **(A)** The optical configuration of imaging when an aberration was introduced into the system by a curved surface. Images of the **(B)** point spread function, **(C)** object intensity pattern and **(D)** reconstructed image. **(E)** Optical configuration when RAP was created on a flexible insulation tape and attached to a curved surface and implemented. Images of the **(F)** point spread function, **(G)** object intensity pattern and **(H)** reconstructed image.

Section S7. Axial resolution of an imaging system using chaotic waves

The axial resolution of RAP was simulated for u = 10 cm, v = 10 cm, D = 2 mm, λ = 0.6 µm and n = 100, where n is the number of pinholes, for pinhole radius R = 10 µm, 20 µm, 30 µm, 40 µm and 50 µm. The axial resolution of a direct imaging system is given by $\sim 8\lambda(u/D)^2$=12 mm and the width of the central maxima is $16\lambda(u/D)^2$ = 24 mm. The FWHM of the axial intensity variation is $\sim 6.7\lambda(u/D)^2$ = 10 mm. The intensity at the origin is observed for direct imaging during blurring of the image in the sensor plane when the object is moved from u = 5 cm to 15 cm. The plots of the variation of the value at the origin for the above values of pinhole diameters for indirect imaging and for direct imaging are shown in **Fig. S14**. The plots indicate the improvement in axial resolving power with a decrease in the diameter of the pinhole approaching the axial resolution limit of direct imaging. The FWHM of the plots were calculated for R = 10 µm, 20 µm, 30 µm, 40 µm and 50 µm and plotted as shown in **Fig. S15**. An approximate semi-empirical relationship has been established between the radius of the pinholes and the FWHM of the axial intensity variation as $\Delta_{a\text{-}FWHM}(R) \sim 6.7\lambda(u/D)^2 + K_1\times(R\text{-}10)$ for $R \geq 10$ µm, and the proportionality factor K_1=33×10^{-5} is the slope of the variation of the FWHM of the axial intensity to the radius of the pinhole.

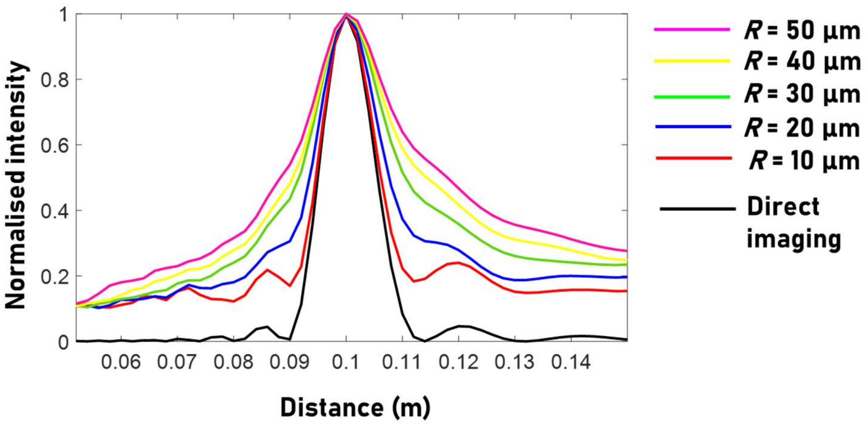

Fig. S14 Plot of the variation of *I*(*x*=0, *y*=0) with the object distance *u*. The radius of the pinhole was varied R = 10 µm, 20 µm, 30 µm, 40 µm and 50 µm.

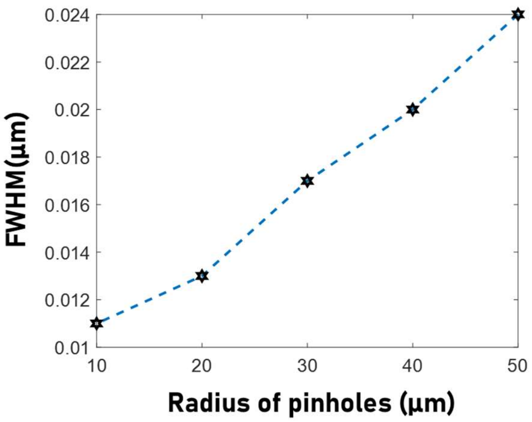

Fig. S15 FWHM of variation of *I*(*x*=0,*y*=0) vs Radius of pinholes. The radius R of the pinholes was varied from 10 µm to 50 µm in steps of 10 µm and the corresponding FWHM were calculated and plotted.

Section S8. Spectral resolution of an imaging system using chaotic waves

The spectral resolution of a sequential imaging system depends upon the resolution of the spectral scanning system installed either at the source or at the detector. Three cases are considered for the same optical configuration: refractive lens, diffractive lens and RAP. The following design parameters are considered for the comparison. u = 10 cm, v = 10 cm, D = 2 mm, λ = 0.4-0.8 µm (0.6 µm as design wavelength), f = 5 cm and n = 100. A refractive lens made up of silica is considered. The dispersion formula given by the Sellmeier equation for silica is given as $\mu^2 = 1 + \frac{B_1\lambda^2}{\lambda^2 - C_1} + \frac{B_2\lambda^2}{\lambda^2 - C_2} + \frac{B_3\lambda^2}{\lambda^2 - C_3}$, where the Sellmeier coefficients for silica are B_1= 0.6961663, B_2= 0.4079426, B_3= 0.8974794, C_1= 0.0046791 µm^2, C_2= 0.0135120 µm^2, C_3= 97.934002 µm^2 [6]. The plot of the refractive index for different wavelengths is shown in **Fig. S16**. The refractive index of silica for λ = 0.6 µm is 1.458. The radius of curvature of a biconvex lens using Lens maker's formula is given as R=2f(μ-1) = 4.58 cm. The focal length is plotted with respect to the wavelength λ = 0.4-0.8 µm in the **Fig. S16**. The radius of zones of diffractive lens is given as $r_n \cong \sqrt{2nf\lambda}$ and so $f \cong \frac{r_n^2}{2n\lambda}$. The radius of the first zone for λ = 0.6 µm and f = 5 cm is given as r_1= 245 µm. The focal length $\left(\frac{6\times10^{-8}}{2\lambda}\right)$ is plotted as a function of wavelength in **Fig. S16.** From the plots it is seen that the diffractive lens has a higher chromatic aberration in comparison to a refractive lens.

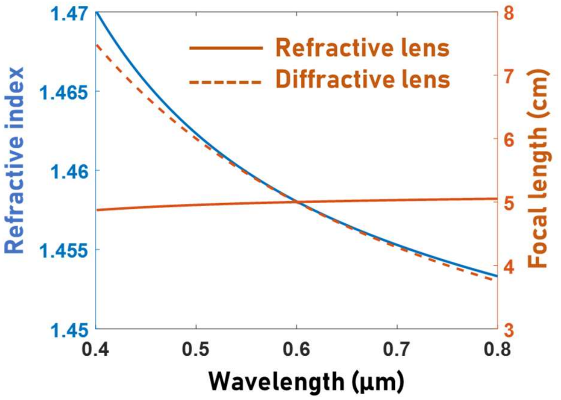

Fig. S16 Refractive index and focal length variation for refractive and diffractive lenses. Plot of the refractive index (blue) and focal length of refractive lens (brown-solid line) and diffractive lens (brown-dotted line) as a function of wavelength λ = 0.4-0.8 µm.

A delta-like function is imaged using the three elements namely refractive lens, diffractive lens and a RAP and the wavelength is varied from 0.4-0.8 µm. The variation of the central intensity value as a function of wavelength is plotted in **Fig. S17.**The spectral resolution dependency on the diameter of the pinholes is studied. $I_{PSF}(\lambda = 600\text{ nm})$ is calculated and cross-correlated with $I_{PSF}(\lambda)$ when the wavelength is varied from λ = 401 nm to 800 nm in steps of 1 nm. The plot of the $I_R(x=y=0)$ for different wavelengths and for different radii of the pinholes R = 10 µm to 50 µm in steps of 10 µm are shown in **Fig. S17**.

From the plots, it is seen that the spectral resolutions improve when the diameter of the pinholes decreases and matches with that of a diffractive lens when $R \leq 10$ µm. The spectral resolution of a refractive lens made of silica has the least spectral resolution. Therefore, for a direct imaging system with a refractive lens, it is almost impossible to resolve images based on wavelength without a spectral scanning system. For the same reason, the direct imaging system does not suffer from chromatic aberrations as a diffractive imaging system does. The ability

for chromatic aberration forms the basis for wavelength discrimination and multidimensional imaging without a colour camera.

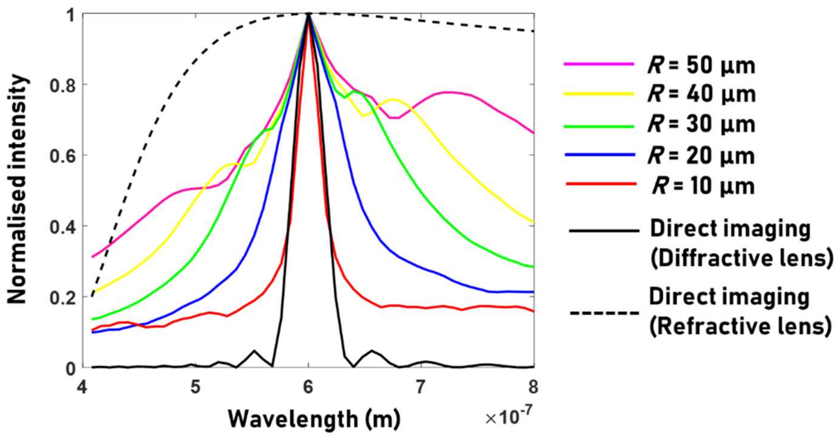

Fig. S17 Spectral sensitivity of direct imaging with refractive lens and diffractive lens and RAP. Plot of the $I_R(x=y=0)$ for λ = 401 nm to 800 nm for R = 10 µm to 50 µm in steps of 10 µm for RAP and direct imaging using a refractive and diffractive lens.

Section S9. Depth-Wavelength relationships

Quasi-achromatic lenses with capabilities to change focal distance by changing wavelength wavelength have been investigated [7]. Let us consider Eq. (S.1), in which the distance and wavelength factors which affect I_{PSF} are λu and λv in the quadratic and linear phase factors. Therefore, any change in the wavelength can be compensated by an equal and opposite change in the distances. For instance, if the wavelength is increased by a factor of k, then if the distances u and v are decreased by a factor of $1/k$, the function I_{PSF} remains a constant. Consequently, I_{PSF} for a wavelength λ_1 can be synthesized from a different wavelength λ_2 by varying u and v by a factor of λ_1/λ_2. On the other hand, by varying λ and v, it is possible to obtain I_{PSF} for a different u. This is an extraordinary relationship as it is possible to discriminate wavelength without having all the wavelength samples and discriminate depth without having all the depth sampled using the point object. A computer simulation was carried out to validate the above idea using the following parameters: u = 10 cm, v = 10 cm and λ_1 = 617 nm. The wavelength was varied to λ_2 = 530 nm.

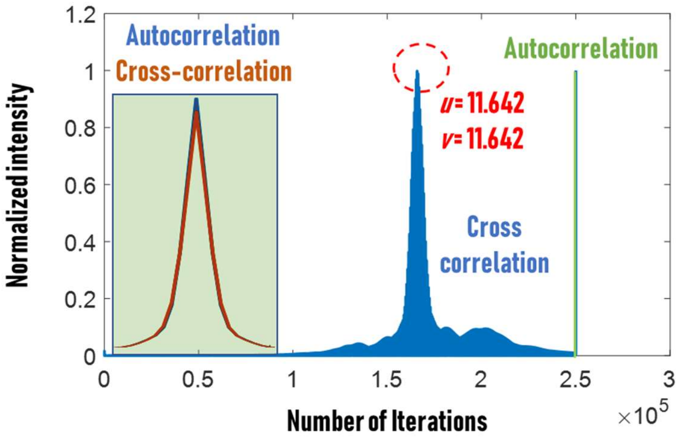

Fig. S18 Plot of the normalized cross-correlation intensity for varying *u* and *v* values. The normalized cross-correlation intensity I_R ($x = 0$, $y = 0$) between $I_{PSF}(u = 10$ cm, $v = 10$ cm, $\lambda_1 = 617$ nm) and $I_{PSF}(u = u_1, v = v_1, \lambda_2 = 530$ nm) for different values of u and v and autocorrelation of $I_{PSF}(u = 10$ cm, $v = 10$ cm, $\lambda_1 = 617$ nm). The inset shows the plot of the autocorrelation (blue) and cross-correlation (orange) graphs.

The values of u and v were iterated between 5 cm and 15 cm in steps of 20 μm to find a case $u = u_1$ and $v = v_1$ when the cross-correlation between $I_{PSF}(u = 10$ cm, $v = 10$ cm, $\lambda_1 = 617$ nm) and $I_{PSF}'(u_1, v_1, \lambda_2 = 530$ nm) equals the autocorrelation of $I_{PSF}(u = 10$ cm, $v = 10$ cm, $\lambda_1 = 617$ nm). The simulation result of the $I_R(x = 0, y = 0)$ for different value of u and v is plotted in **Fig. S18**. As expected, the cross-correlation value matched with the autocorrelation for a case $u = u_1$ and $v = v_1$ and $\lambda_2 = 530$ nm and the values of $u_1 = v_1 = 11.642$ cm. The factor of increase of u_1 and v_1 is the same as the factor of decrease of λ_1 to λ_2. The above finding reduces the spectral and spatial sampling requirements.

Section S10. Computational modules for automation

The computational module was developed using open source components namely Debian GNU/Linux (Ubuntu 16.04) as an operating system and GNU Octave (4.3.0) for video processing. For easy implementation, this module has been presented to be used with any available web camera. The schematic of the computational module is shown in **Fig. S19**.

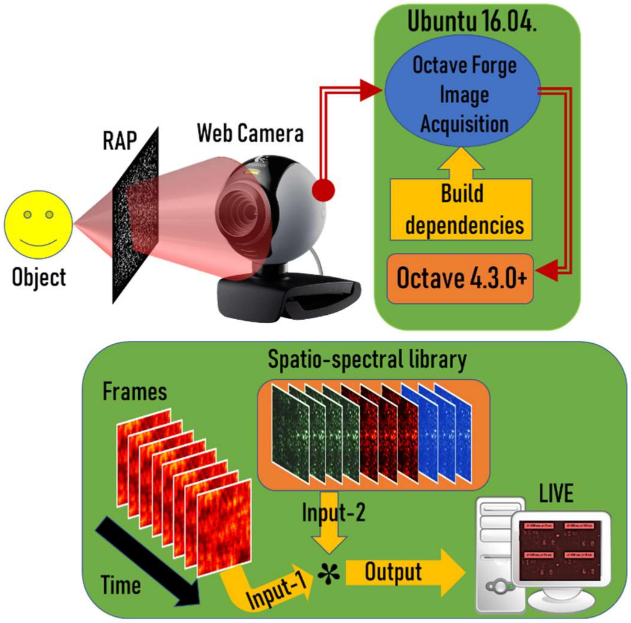

Fig. S19. **Assistive computational modules.** Schematic of the computational module for real-time seeing of events synchronously in different spatio-spectral dimensions.

The spatio-spectral signatures are recorded by sampling along different wavelengths and depths which are catalogued and stored in a library. The software grabs one frame at a time and cross-correlates the frame using an optimal non-linear filter with the spatio-spectral signatures and reconstruct the frame simultaneously in different spatio-spectral dimensions. This process is repeated for different frames and the corresponding reconstructions are updated in the computer display. The Octave code for building dependencies and automation is given in **Database file**.

Section S11. Octave code for automation

```
##?GNU Octave, version 4.4.1
##Copyright (C) 2018 John W. Eaton and others.
##This is free software; see the source code for copying
conditions.
##There is ABSOLUTELY NO WARRANTY; not even for
MERCHANTABILITY or
##FITNESS FOR A PARTICULAR PURPOSE. For details, type
'warranty'.
##
##Octave was configured for "x86_64-w64-mingw32".
##
##Additional information about Octave is available at
https://www.octave.org.
##
##Please contribute if you find this software useful.
##For more information, visit https://www.octave.org/get-
involved.html
##
##Read https://www.octave.org/bugs.html to learn how to
submit bug reports.
##For information about changes from previous versions,
type 'news'.
%This is a simple guide for automation of the 5d imaging
system involving
grabbing the feed from the web camera frame by frame and
implementing the
cross-correlation in real-time and display the
correlation result real
time.
% The installation guide has been developed based on the
computer used for
% the automation. From our experience, we found that the
dependecies
% requirement vary with the version of the operating
system and other
% software. There is absolutely no warranty to the
installation guide.
% Step - 1 Install GNU Linux Ubuntu 16.04.
(http://releases.ubuntu.com/16.04/)
% Step - 2 Update Ubuntu using the command : 'sudo apt-
get update and sudo
apt-get install build-essential' (Terminal application).
% Step - 3 Build dependencies for Image-acquisition
package of Octave.
```

```
'''libv4l-dev''' and '''libfltk1.3-dev''' or
'''libfltk1.1-dev'''.
%It can be installed on GNU/Linux in ubuntu 16.04 using
the command: 'sudo
apt install libv4l-dev libfltk1.3-dev -y'
% Step - 4 Build dependencies liboctave-dev,libavutildev,
libswscale,libswscale-de,libavcodec-
dev,FFmpeg,libavformat-dev for
addframe, avifile, aviinfo and aviread for saving the
feed from the image
sensor into a video file.
%It can be installed on GNU/Linux in Ubuntu 16.04 using
the command: 'sudo
apt-get install liboctave-dev,libavutil-
dev,libswscale,libswscalede,
libavcodec-dev,FFmpeg,libavformat-dev -y'
% Step - 5 Install GNU Octave
(https://www.gnu.org/software/octave/download.html) and
install Octave
forge packages namely image, video and image-acquisition.
##https://octave.sourceforge.io/image/
##https://octave.sourceforge.io/video/index.html
##(https://octave.sourceforge.io/image-
acquisition/index.html)
% Step - 6 For installation of image-acquisition
## (a) hg clone http://hg.code.sf.net/p/octave/image-
acquisition
## (b) Move to src directory (cd image-acquisition/src/)
and run the
command:
## './configure'
##after successful run,
##'make'
##and
##'make install'
%% Check the name of the webcamera device
%% In terminal window type 'v4l2-ctl --list-devices'
%% This will show the camera type and the names assigned
##iball face2face webcamera
## /dev/video3
pkg load image-acquisition% Loads the image-acquisition
package
pkg load video% Loads the video package
% Reference
x=videoinput("v4l2","/dev/video3"); %Open the v4l2 device
set(x, "VideoFormat", "RGB3");
set(x, "VideoResolution", [640 480]);
```

```
start(x)
img1=getsnapshot(x);% Grabs a spatio-spectral signature
before starting the
event
imwrite(img1,'img1.jpg');% Save the image in the computer
Reference=double(img1);% Coverts image into a matrix with
floating values
Reference1=Reference(:,:,1); % Extracts a single channel
Reference2=zeros(960,1280); % Create a matrix twice the
size of the image
Reference2(241:720,321:960)=Reference1; % Zeropad the
recorded data
stop(x)
% Sample
x=videoinput("v4l2","/dev/video3"); %Open the v4l2 device
set(x, "VideoFormat", "RGB3");
set(x, "VideoResolution", [640 480]);
start(x)
m = avifile("test1.avi", "codec", "msmpeg4v2")% Open
videofile for writing
frame by frame
o = avifile("test2.avi", "codec", "msmpeg4v2")% Open
videofile for writing
frame by frame
for i=1:100;
img2=getsnapshot(x);% Grabs an image after start of the
event
imwrite(img2,'img2.jpg');% Save the image in the computer
Sample=double(img2);% Coverts image into a matrix with
floating values
Sample1=Sample(:,:,1);% Extracts a single channel
Sample2=zeros(960,1280);% Create a matrix twice the size
of the image
Sample2(241:720,321:960)=Sample1;% Zeropad the recorded
data
sam=Sample1/max(max(Sample1));% Normalize (0-1)
A=fft2(Reference2);
alpha=0;% optimal alpha value
beta=0.6;%optimal beta value
A1=abs(A).^alpha;
A2=exp(1i*arg(A));
B=fft2(Sample2);
B1=abs(B).^beta;
B2=exp(1i*arg(B));
Object=ifftshift(ifft2(A1.*A2.*conj(B1.*B2)));
R=abs(Reconstruction);%Calculate the absolute of the
reconstructed image
```

```
R=R/max(max(R));% Normalize (0-1)
imagesc(R); % Display reconstructed image real-time
pause(0.1) % pause
addframe(m, R) % Write a frame to the video file of
reconstructed image
addframe(o, sam) % Add frame to the video file
printf(".")
end
printf("\n")
stop(x) % End recording
```

References

1. M. Kumar, A. Vijayakumar and J. Rosen, "Incoherent digital holograms acquired by interferenceless coded aperture correlation holography system without refractive lenses" *Sci. Rep.* **7**, 11555 (2017).
2. J. C. Dainty, *Laser speckle and related phenomena*. **9** (Springer science & business Media, 2013).
3. X-B. Hu, M-X. Dong, Z-H. Zhu, W. Gao, and C. Rosales-Guzmán. "Does the structure of light influence the speckle size?." *Sci. Rep.* **10**, 1 (2020).
4. R. Rai, A. Vijayakumar and J. Rosen, "Non-linear Adaptive Three-Dimensional Imaging with interferenceless coded aperture correlation holography (I-COACH)" *Opt. Express* **26**, 18143 (2018).
5. J. L. Horner and P. D. Gianino, "Phase-only matched filtering," *Appl. Opt*. **23**, 812-816 (1984).
6. G. Ghosh, "Sellmeier coefficients and dispersion of thermo-optic coefficients for some optical glasses," *Appl. Opt*. **36**, 1540-1546 (1997).
7. A. Vijayakumar and S. Bhattacharya, "Quasi-achromatic Fresnel zone lens with ring focus," *Appl. Opt.* **53**, 1970-1974 (2014).
